# Protein Spin Labeling with a Photocaged Nitroxide Using Diels–Alder Chemistry†

**DOI:** 10.1002/cbic.201900318

**Published:** 2019-08-14

**Authors:** Anandi Kugele, Bjarne Silkenath, Jakob Langer, Valentin Wittmann, Malte Drescher

**Affiliations:** ^1^ Department of Chemistry and Konstanz Research School Chemical Biology (KoRS-CB) University of Konstanz Universitätsstrasse 10 78457 Konstanz Germany

**Keywords:** bioorthogonal chemistry, cycloaddition, EPR spectroscopy, noncanonical amino acids, site-directed spin labeling

## Abstract

EPR spectroscopy of diamagnetic bio‐macromolecules is based on site‐directed spin labeling (SDSL). Herein, a novel labeling strategy for proteins is presented. A nitroxide‐based spin label has been developed and synthesized that can be ligated to proteins by an inverse‐electron‐demand Diels–Alder (DA_inv_) cycloaddition to genetically encoded noncanonical amino acids. The nitroxide moiety is shielded by a photoremovable protecting group with an attached tetra(ethylene glycol) unit to achieve water solubility. SDSL is demonstrated on two model proteins with the photoactivatable nitroxide for DA_inv_ reaction (PaNDA) label. The strategy features high reaction rates, combined with high selectivity, and the possibility to deprotect the nitroxide in *Escherichia coli* lysate.

## Graphical abstract

**PaNDA proteins**: The photoactivatable nitroxide for DA_inv_ reaction (PaNDA; DA_inv_: inverse‐electron‐demand Diels–Alder) is a novel spin label for proteins. This newly developed nitroxide‐based spin label can be ligated to proteins through a DA_inv_ cycloaddition to genetically encoded noncanonical amino acids.

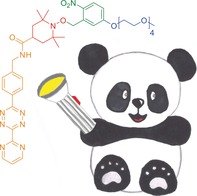

EPR spectroscopy together with site‐directed spin labeling (SDSL) is a valuable and established tool to elucidate structure, function, and dynamics of proteins and protein complexes.^1^ Nitroxide‐based spin labels are the best established and most convenient ones,^2^ because they are small, nondisturbing, and exhibit excellent spectroscopic properties.^3^ In particular, they are perfect to display rotational dynamics through line‐shape analysis3a, 4 and can be used for distance determination.^5^ The most commonly used spin label for EPR spectroscopy is the methanethiosulfonate spin label (MTSSL).1b It reacts specifically with accessible sulfhydryl moieties in proteins, that is, cysteine residues. MTSSL application requires elimination of native cysteine residues by genetic engineering and the introduction of strategically positioned residues. Alternative native amino acids, such as tyrosine, were also utilized for spin labeling.^6^ However, the choice of native amino acids as tags for spin labels limits bioorthogonality. If selecting genetically encoded noncanonical amino acids (ncAAs) as targets for SDSL instead, selectivity can be achieved, even in cells, and removal of functionally relevant cysteine residues is not required.^7^ The use of an orthogonal aminoacyl‐tRNA‐synthetase (aaRS)–tRNA pair capable of selectively charging a nonsense suppressor tRNA (e.g., an amber codon) with an ncAA is an established method.^8^ Expansion of the genetic code by the incorporation of ncAAs has resulted in a plethora of potential conjugation techniques because a broad range of ncAAs with specific reactivities are available.^9^

However, in combination with spin labeling, only a few ncAAs and corresponding chemical reaction schemes have been employed, to date.^10^ An ideal spin labeling procedure should exhibit high reaction rates, but still be selective. Independence from any potentially cytotoxic catalysts further simplifies the reaction, while water solubility is a prerequisite for in vivo use. Gadolinium(III)‐ and trityl‐based spin labels are stable in cells. They have been used for distance measurements, but suffer either from a very broad spectrum and resulting low modulation depths or from a very narrow spectrum not suitable for double‐frequency experiments.10b, 11 Nitroxides provide ideal spectral width and additionally access to dynamic information. However, traditional nitroxide‐based labels feature limited redox stability in the cellular environment and their EPR signal vanishes within minutes.^12^ Thus, for routine in vivo use, it is crucial to increase the nitroxide stability. Recently, a tetraethyl‐modified maleimido‐PROXYL‐based (PROXYL: 2,2,5,5‐tetramethyl‐1‐pyrrolidinyloxy) spin label with enhanced stability was introduced.^13^

We present herein a novel approach to address both ncAA‐mediated spin labeling and nitroxide stability (Figure 1). Introduction of the novel label is achieved through the DA_inv_ reaction^14^ of a 1,2,4,5‐tetrazine with a strained alkyne (cyclooctyne, SCO)^15^ or alkene (*trans*‐cyclooctene, TCO).^16^ This reaction forms the corresponding pyridazine and dihydropyridazine, respectively. Due to its excellent water compatibility, the DA_inv_ reaction has proven to be suitable for a broad range of biochemical applications both in vitro and in vivo.^14^, ^17^ Strain‐promoted DA_inv_ cycloadditions have not been used for the SDSL of proteins so far (notably, recently an in vitro transcribed RNA segment was site‐specifically labeled with a tetrazine–nitroxyl moiety^18^). Moreover, we aimed to address nitroxide stability by using a protection strategy. So far, alkylation,^19^ silylation,^20^ acylation,^21^ and photoremovable protecting groups (PPGs)^22^ have been demonstrated to protect nitroxides and to release them as needed. In particular, photoirradiation for deprotection is interesting because it enables spatial and temporal control over the release of functional groups. In particular, *o*‐nitrobenzyl derivatives for diverse functionalities, even for native amino acid side chains,^23^ were pioneered in 1966 and have since become the best established PPGs.^24^ Their application for the protection of nitroxide spin labels during oligonucleotide synthesis was introduced by Seven et al. in 2014^22^ and continued by Weinrich et al.^25^

**Figure 1:**
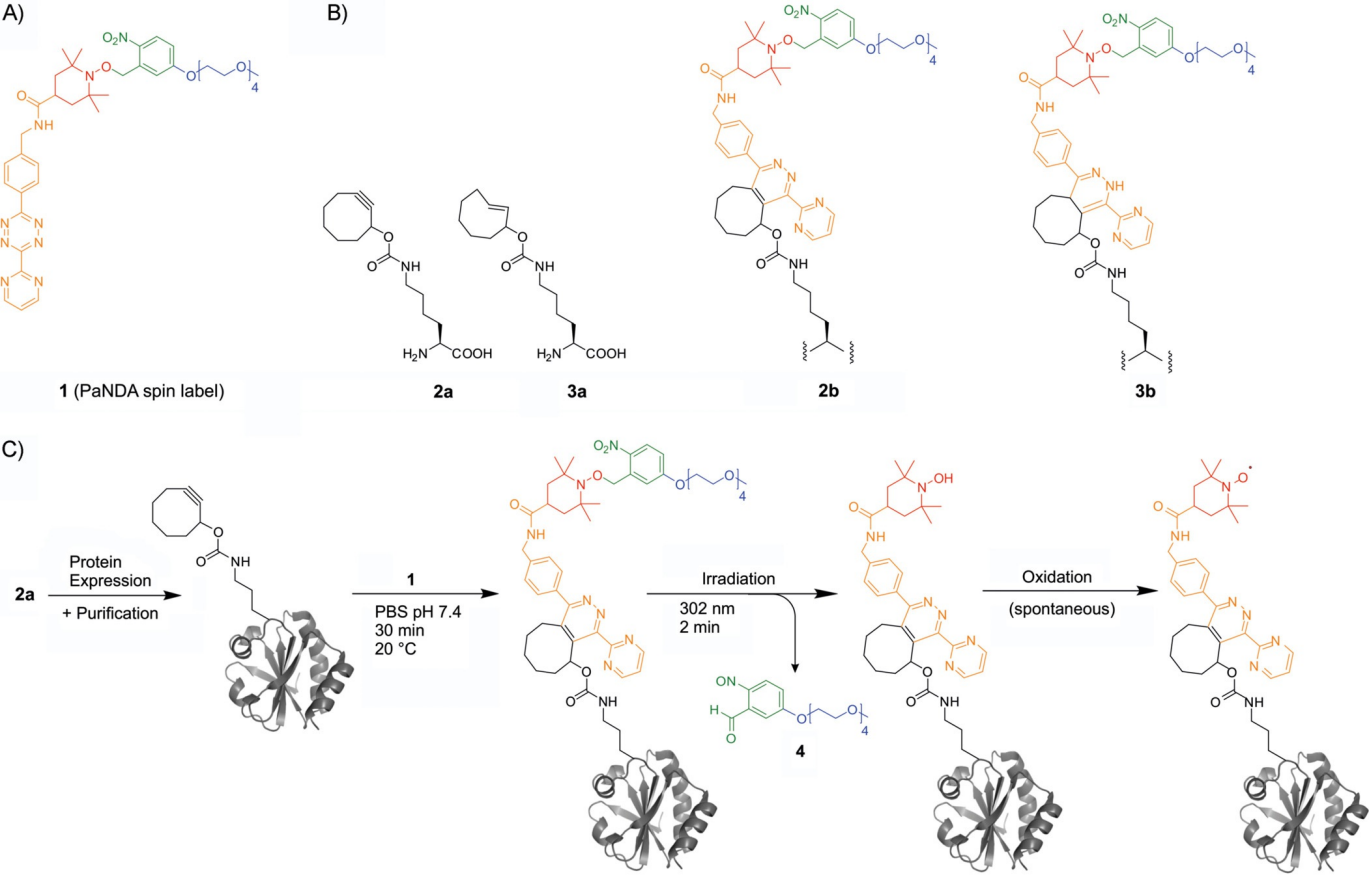
Protein labeling with the photoactivatable nitroxide for DA_inv_ reaction (PaNDA; DA_inv_: inverse‐electron‐demand Diels–Alder) label. A) Structure of PaNDA spin label **1**, with its particular functional features colored in orange (tetrazine moiety), red (photocaged (2,2,6,6‐tetramethylpiperidin‐1‐yl)oxyl (TEMPO)), and green (photoremovable protecting group (PPG), with a tetra(ethylene glycol) chain in blue). B) Structures of the ncAAs *N* ^6^‐[(cyclooct‐2‐yn‐1‐yloxy)carbonyl]‐l‐lysine (**2 a**) and *N* ^6^‐({[(*E*)‐cyclooct‐2‐en‐1‐yl]oxy}carbonyl)‐l‐lysine (**3 a**) used in this study. The respective products upon reaction with **1** are shown as **2 b** and **3 b** for the ncAAs **2 a** and **3 a**, respectively. C) Incorporation of the ncAA into proteins (exemplarily shown for TRX‐R74→**2 a** (PDB ID: 2TRX^[27]^)), and in vitro labeling and deprotection conditions. PBS: phosphate‐buffered saline.

Figure 1 A depicts the structure of the new spin label, named PaNDA. It is composed of a 3,6‐diaryl‐substituted 1,2,4,5‐tetrazine as the diene component of a DA_inv_ reaction attached to an *o*‐nitrobenzyl‐protected TEMPO derivative, which delivers an EPR‐active nitroxyl radical upon photoirradiation and subsequent air oxidation. Oligoethylene glycol chains have been used previously to enhance the water solubility of tetrazine derivatives.^26^ Accordingly, we attached a tetra(ethylene glycol) monomethyl ether to ensure water solubility of the PaNDA label. This approach is elegant, insofar as the rather bulky PPG, along with the tetra(ethylene glycol) chain, is cleaved off by irradiation.

For the synthesis of PaNDA label **1**, carboxy TEMPO **5** was quantitatively transformed into hydroxylammonium salt **6** by making use of the acid‐dependent redox triad of a nitroxyl radical, an oxoammonium cation, and hydroxylamine (Scheme 1 A).^28^ Conveniently, the carboxylic acid is simultaneously protected under these conditions. Nitrobenzaldehyde **7** was reduced to the benzyl alcohol and the phenol group was selectively alkylated with tetraethylene derivative **8** to yield nitrobenzyl alcohol **9** (Scheme 1 B). The Appel reaction gave benzyl bromide **10**, which was used to alkylate **6** followed by ester hydrolysis to yield carboxylic acid **11**. Finally, amide bond formation with tetrazine derivative **12** 29 delivered PaNDA label **1** in an overall yield of 42 %, starting from nitrobenzaldehyde **7**.

**Scheme 1:**
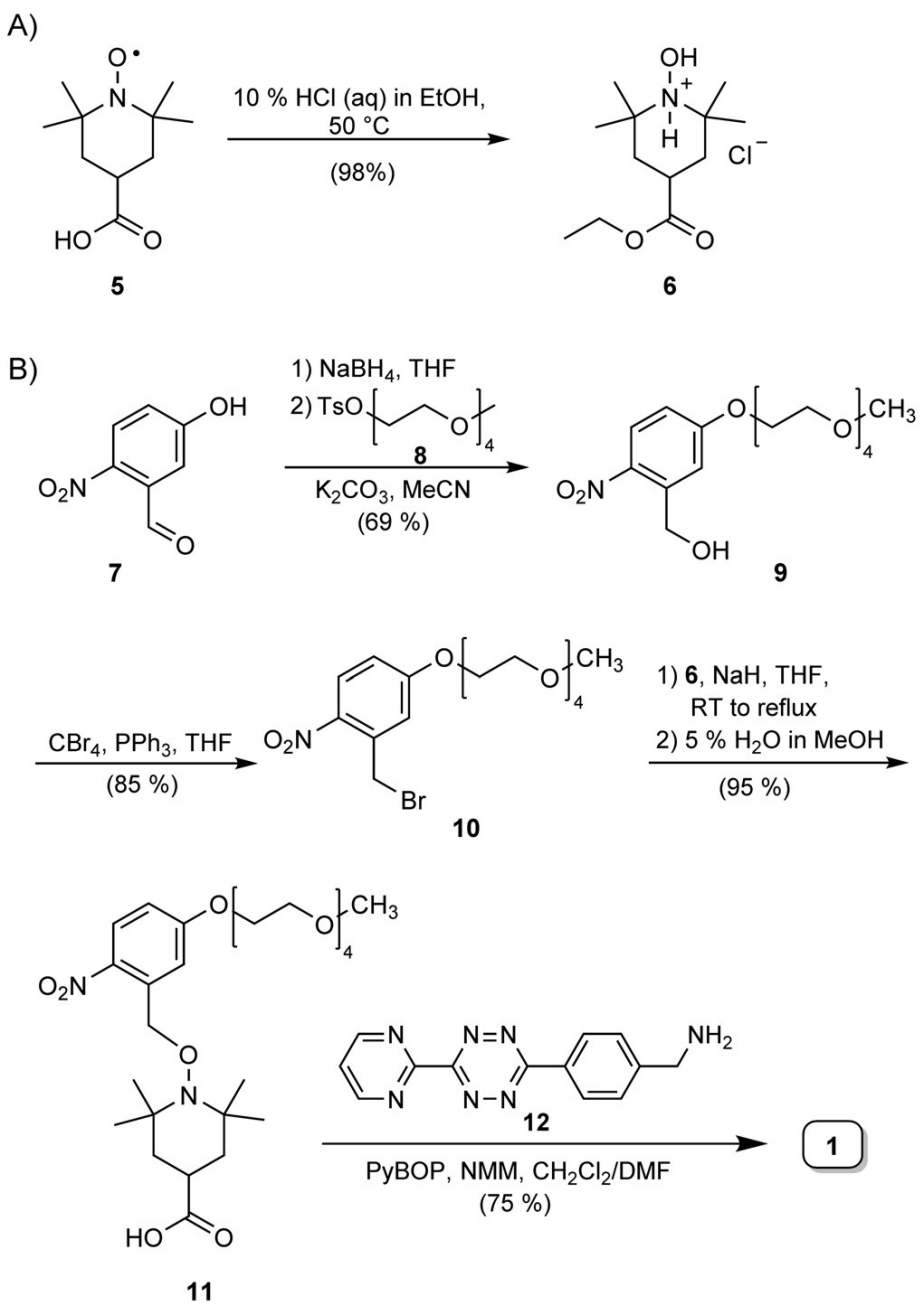
Synthesis of PaNDA label **1**. Ts: toluenesulfonyl, NMM: *N*‐methylmorpholine, PyBOP: benzotriazol‐1‐yloxytripyrrolidinophosphonium hexafluorophosphate.

To initially test and optimize the deprotection step, we evaluated the UV/Vis absorption spectrum of the PaNDA spin label (Figure 2 A). It exhibits a prominent feature for the photocage group at *λ*≈300 nm. To this end, we tested the time‐dependent deprotection efficiency at *λ*=302 nm. LC‐MS analysis of the PaNDA spin label proved almost complete conversion to the desired nitroxide after irradiation for 10 min (Figure 2 B). Accordingly, an EPR signal appeared only after irradiation (Figure 2 C).

**Figure 2:**
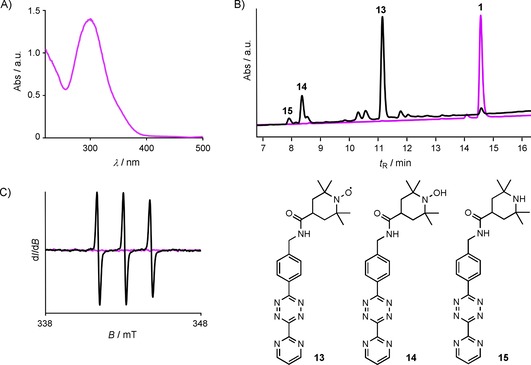
Deprotection study of the PaNDA spin label **1**. A) UV absorption spectrum of 75 μm **1** in methanol. B) LC‐MS traces showing the absorption at *λ*= 254 nm of **1** before (purple) and after irradiation (black) for 10 min at *λ*=302 nm. After irradiation, label **1** is virtually completely consumed and nitroxide **13** is the main product. Small amounts of intermediate **14** and side product **15** are visible. C) EPR spectrum of 100 μm **1** in water before (purple) and after irradiation (black).

To test and establish our labeling and deprotection method with proteins, we chose green fluorescent protein (GFP) and *Escherichia coli* oxidoreductase thioredoxin (TRX). TRX was the first protein to be modified by a DA_inv_ reaction in 2008,16a and previous EPR studies are available for data comparison.10e, 30 Both model proteins exhibit native cysteine residues; thus providing the possibility to test for orthogonal labeling without affecting cysteine. The first ncAA that could undergo a DA_inv_ reaction, a lysine‐derived cyclooctyne, was introduced for the copper‐free click reaction in 2011,^31^ before a tetrazine,^32^ norbornenes,15a, 33 TCOs,15a and a spirohexene^34^ were also genetically encoded. The rationally designed *Methanosarcina mazei* mutant tRNA^Pyl^/PylRS^AF^ (Y306A, Y384F) possesses an enlarged binding pocket that is suitable for incorporating these bulky ncAAs in response to the amber stop codon.15a, 31, 35 Therefore, we coexpressed amber stop codon mutants of C‐terminally His_6_‐tagged model proteins, together with the previously reported tRNA^Pyl^/PylRS^AF^ synthetase in *E. coli*. We tested two different ncAAs, namely, **2 a** and **3 a** (Figure 1 B), which are well known for high integration rates and exhibit excellent reaction kinetics.15a, 36 Expression in the presence of 1 mm ncAA yielded between 4 and 7 mg L^−1^ of purified full‐length GFP‐Y39→**2 a**, GFP‐Y39→**3 a**, TRX‐R74→**2 a**, or TRX‐R74→**3 a**, respectively (Figures S11–S12 and S16–S17 in the Supporting Information). We even succeeded in the incorporation of the SCO‐bearing amino acid **2 a** in response to two amber stop codons in a protein, which was, to the best of our knowledge, not reported previously (TRX‐G34/R74→**2 a**; Figure S13).

Next, we wanted to assess the spin labeling potential of the ncAA‐containing proteins with the PaNDA spin label in vitro (for the general procedure, see Figure 1 C). For this purpose, we mixed the purified proteins with the PaNDA spin label and established mild and universally applicable reaction conditions (30 min, 20 °C). We separated the labeled proteins from excess label by using Ni‐NTA resin, and measured EPR spectra before and after irradiation (Figure 3). Data recorded before irradiation indicate that the PPG is stable enough to survive the labeling and washing procedure. EPR spectra after irradiation show that both model proteins comprising **2 a** or **3 a** were successfully labeled and deprotection worked. Spectra of the wt proteins incubated with the PaNDA spin label exhibit no EPR signal; this indicates high chemoselectivity of this reaction (Figure 3, upper line).

**Figure 3:**
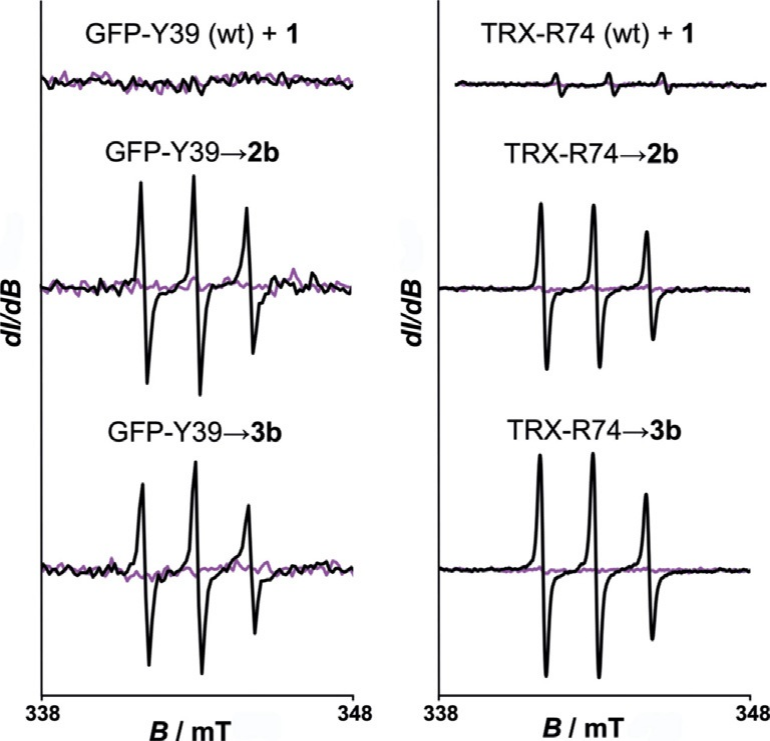
SDSL of proteins by DA_inv_ cycloaddition and subsequent deprotection of the PPG. EPR spectra of GFP and TRX bearing amino acids, as indicated (wild type (wt) shown in the first row) and reacted with the PaNDA spin label after purification. Spectra are shown before (purple) and after (black) irradiation. The signal intensities are drawn on the same scale for all spectra in one column. Approximately 1 μm spin concentration for TRX‐R74 (wt)+**1** indicates minor remaining impurities of unreacted PaNDA after purification.

By means of circular dichroism spectroscopy, we further proved that the labeled proteins kept their secondary structure throughout the process (Figure S20). By analyzing the double integral of the EPR spectra, we found that deprotection of the PaNDA spin label was maximal after 2 min of irradiation at *λ*=302 nm (Figure S21). To substantiate our data, full‐length ESI‐MS spectra were recorded (Figures S18 and S19). Signals can be assigned to successfully labeled and deprotected protein, but no mass signals were found that corresponded to still protected protein–label complex (occurrence of signals with *Δ*=−150 Da for **2 a** or **2 b**, or *Δ*=−152 Da for **3 a** or **3 b** indicated partial degradation of the ncAA to lysine). Thus, the design of our spin label allows for fast and selective labeling, as well as convenient and quantitative deprotection.

We assessed the performance of our protection strategy in biological environments. We reasoned that reducing conditions could potentially intervene with the spontaneous oxidation step, converting the irradiation‐derived hydroxylamine into a nitroxide.^22^ To this end, we chose *E. coli* lysate because it contained relevant cell components and provided reducing conditions. We first labeled TRX‐R74→**2 a** with the PaNDA spin label and deprotected the obtained TRX‐R74→**2 b** as described above. Then we incubated the lysate with this protein to confirm nitroxide degradation. After 80 min, the EPR signal was <1 μm (Figure 4). Then, we incubated labeled and still protected TRX‐R74→**2 b** with the same amount of fresh *E. coli* lysate for 80 min. No EPR signal was detected in this period of time; this meant that the protecting group remained stable on the protein. After irradiation, the EPR signal increased; this resulted in approximately 50 % spin concentration compared with deprotection in buffered solution.

**Figure 4:**
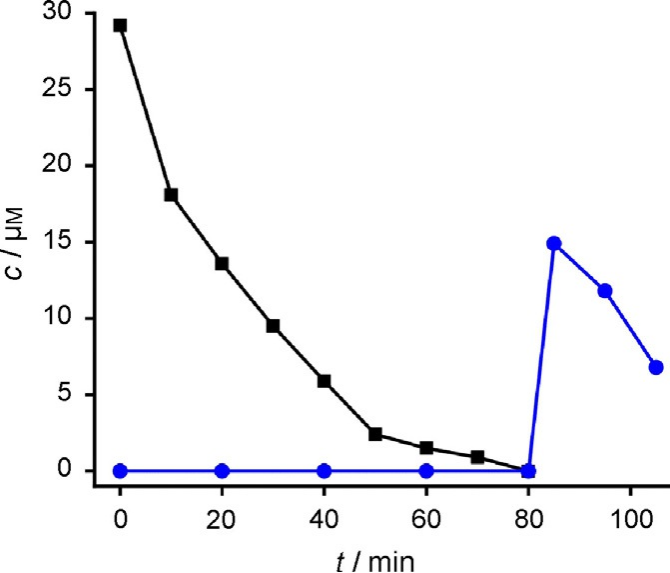
Recovery of nitroxide radicals is possible in *E. coli* lysate. *E. coli* cells were lysed and mixed with preliminarily irradiated TRX‐R74→**2 b** (▪) and the spin concentration was quantified at different time points until the signal had vanished (<1 μm) after 80 min. In comparison, a nonirradiated sample of TRX‐R74→**2 b** (•) was incubated with lysate for 80 min, showing no EPR signal. After irradiation for 2 min at *λ*=302 nm, a spin concentration of approximately 15 μm was found (see Figure S22 for EPR spectra at each time point).

This demonstrates that a spontaneous oxidation step to form the nitroxide is actually possible in a reducing environment, and that, in principle, deprotection can be performed in *E. coli* lysate to recover the EPR signal.

We envisage the application of the PaNDA spin label for in‐cell EPR measurements. Points that need to be addressed before the system can be used in cells involve the delivery of the PaNDA spin label into cells and removal of unbound label. Moreover, labeling and deprotection efficiencies need to be confirmed in cells. However, the DA_inv_ reaction, in general, is known to be well suited to bioorthogonal intracellular reactions.^37^ Moreover, *o*‐nitrobenzyl and other PPGs were shown to be cleaved upon irradiation, even inside cells.^38^ To address nitroxide lability after irradiation, samples for EPR distance measurements will be frozen directly after being irradiated; thus minimizing nitroxide degradation.

In conclusion, we have developed an innovative SDSL approach based on genetically encoded ncAAs amenable to DA_inv_ chemistry and on a newly designed spin label. The PaNDA spin label provides an appealing combination of a tetrazine moiety and a PPG. The use of DA_inv_ chemistry for spin labeling illustrates the assets and versatility of this reaction. For in‐cell labeling or binding studies in cellular environments, the PPG potentially enables prolonged incubation times before data acquisition. Thus, our method opens up new vistas for the study of proteins by EPR spectroscopy.

## Conflict of interest

*The authors declare no conflict of interest*.

## Supporting information

As a service to our authors and readers, this journal provides supporting information supplied by the authors. Such materials are peer reviewed and may be re‐organized for online delivery, but are not copy‐edited or typeset. Technical support issues arising from supporting information (other than missing files) should be addressed to the authors.

Supplementary
