# Supplementary material for: Protein Spin Labeling with a Photocaged Nitroxide Using Diels–Alder Chemistry
Source: Chembiochem. 2019 Aug 14;20(19):2479–84. doi: 10.1002/cbic.201900318 (PMC6790680; doi:10.1002/cbic.201900318)
Supplement: Supplementary file 1 — Supplementary [file CBIC-20-2479-s001.pdf]

CHEMBIOCHEM

Supporting Information

Protein Spin Labeling with a Photocaged Nitroxide Using Diels–Alder Chemistry**

Anandi Kugele+, Bjarne Silkenath+, Jakob Langer, Valentin Wittmann,* and Malte Drescher*[a]

cbic_201900318_sm_miscellaneous_information.pdf

Table of Contents

Experimental Procedures **S2**

Synthesis of the PaNDA spin label (1) S2

Plasmids and transformation of *E. coli* S7

Expression and purification of TRX and GFP and incorporation of genetically encoded artificial amino acids S7

Site-directed spin labeling of TRX and GFP with the PaNDA spin label S7

Deprotection of the PaNDA spin label by irradiation S8

EPR measurements S8

Full-length mass spectrometry of proteins S8

Measurement of circular dichroism (CD) spectra S8

Experiments with *E. coli* lysate S8

Supporting Figures **S9**

NMR spectra (Figures S1-S10) S9

SDS-PAGE images (Figures S11-S13) S14

Primary sequences of model proteins (Figures S14-S15) S15

Full-length ESI-MS spectra (Figures S16-S19) S16

Further supplementary figures (Figures S20-S22) S20

Supporting References **S22**

Experimental Procedures

Synthesis of the PaNDA spin label (1)

General methods

Technical solvents were distilled prior to use. Dry solvents were either purchased from Sigma Aldrich, Acros or were dried and distilled. For degassing of dry solvents, the freeze-pump-thaw method was applied. Deuterated solvents for NMR spectroscopy were purchased from Deutero.

All reactions were monitored by Thin Layer Chromatography (TLC) with silica gel 60 F_{254} coated on aluminum sheets from Merck. UV active compounds were detected at 254 nm. Additionally, different staining solutions followed by gentle heating were used for the visualization of the reactants (the composition of the solvents is stated as a ratio of volumes (*v*/*v*)):

- Anisaldehyde solution: EtOH (150 mL), acetic acid (15 mL), conc. H_2SO_4 (5 mL), *p*-methoxybenzaldehyde (3.7 mL)
- Ninhydrin solution: EtOH (200 mL), acetic acid (3 mL), ninhydrin (0.2 g)
- Potassium permanganate solution: 0.1 % $KMnO_4$ in 1 M NaOH

For the preparative Flash Chromatography (FC) silica gel 60 (Geduran Si 60, 0.040-0.063 mm particle size) from Merck was used. Solvent mixtures are specified as volume ratio (v/v) and all solvents were distilled prior to usage. Additionally, FC was performed on a MPLC-Reveleris X2 system from Grace.

High Pressure Liquid Chromatography with Mass Detection (LC-MS) analysis was performed with LCMS2020 by Shimadzu (pumps: LC-20 AD, auto sampler: SIL-20A HT, UV-Vis detector: SPD-20A, oven: CTO-20AC, communications bus module: CBM-20A, ESI detector, software LCMS-Solution) with an EC 125/4 C18, 3 µM 40 column (Machery Nagel) and a binary gradient of acetonitrile and water supplemented with 0.1 % formic acid. The flow rate was 0.4 mL*min^{-1}.

Nuclear magnetic resonance (NMR) spectra were recorded on spectrometers Avance III 600 MHz and on Avance III 400 MHz from Bruker at room temperature. The resonance signals of different deuterated solvents were used as internal standards: $CDCl_3$ (δ_H = 7.26 ppm, δ_C = 77.16 ppm), DMSO-d_6 (δ_H = 2.50 ppm, δ_C = 39.5 ppm). In addition to first-order analysis, ^1H, ^1H homo- and ^1H, ^{13}C hetero nuclear two-dimensional correlation spectra like HSQC, COSY, TOCSY and HMBC were recorded for the assignment of signals. $^1J_{H\text{-}1/C\text{-}1}$ couplings were determined from non-decoupled HSQC and HMBC spectra. The multiplicities of the resonances are abbreviated as followed: s (singlet), d (doublet), dd (doublet of doublets), ddd (doublet of doublets of doublets), t (triplet), dt (doublet of triplets), td (triplet of doublets), m (multiplet). The recorded NMR spectra were analyzed by using the software MestReNova v12.0.0-20080 by Mestrelab Research. NMR spectra can be found under the “Results and Discussion” section in Figure S1-S10 of this Supporting Information.

High resolution masses were measured on a micrOTOF II instrument from *Bruker* in positive mode. Electrospray was used as ionization method (ESI) and the time of flight (TOF) method was used for detection. The recorded mass spectra were analyzed by using the software Xcalibur v3.0 by Thermo Fischer Scientific.

*Synthesis of 4-(Ethoxycarbonyl)-1-hydroxy-2,2,6,6-tetramethylpiperidin-1-ium-chlorid (**6**)*

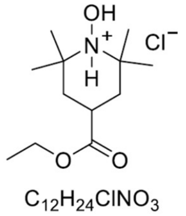

$C_{12}H_{24}ClNO_3$
265.78 g/mol

Carboxy-TEMPO **5** (50 mg, 250 µmol, 1.0 equiv.) was dissolved in EtOH (3 mL) and 37 % $HCl_{(aq)}$ (1 mL). The reaction mixture was stirred at 50 °C for 4 h. The solvent was then evaporated under reduced pressure. The solvent was then

coevaporated with 1 M $HCl_{(aq)}$ (2 x 2 mL). The product **6** was obtained without further purification as a white foam (65 mg, 245 µmol, 98 % yield).

^{1}H NMR (400 MHz, $CDCl_3$, 300 K) δ = 11.52 (s, 1H, N-H), 10.97–10.48 (br. s, 1H, OH), 4.12 (q, 2H, *J* = 7.1 Hz, CH_2-CH_3), 2.79 (tt, 1H, *J* = 13.6 Hz, 3.5 Hz, CH), 2.44 (dt, 2H, *J* = 13.6 Hz, 3.5 Hz, CH_2), 2.05 (dt, 2H, *J* = 13.6 Hz, 3.5 Hz, CH_2), 1.66 (s, 6H, CH_3), 1.38 (s, 6H, CH_3), 1.24 (t, 3H, *J* = 7.1 Hz, CH_3-CH_2).
^{13}C NMR (101 MHz, $CDCl_3$, 300 K) δ = 172.6 (C-carbonyl), 68.0 ($C_{quart.}$), 61.2 (CH_2-CH_3), 38.4 (CH_2), 33.5 (CH), 27.9 (CH_3), 20.4 (CH_3), 14.1 (CH_3-CH_2) ppm.
HRMS (ESI-TOF) calcd. for $C_{12}H_{22}NO_3$ $[M + H]^+$ *m/z* = 230.1751, found: 230.1743.

*Synthesis of 2,5,8,11-Tetraoxatridecan-13-yl-4-methylbenzenesulfonate (**8**)*

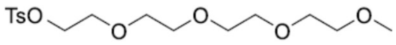

$C_{16}H_{26}O_7S$
362.44 g/mol

The reaction procedure was adopted from Wang et al.[1]

*Synthesis of 3-(Hydroxymethyl)-4-nitrophenol (**16**)*

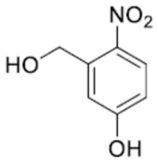

$C_7H_7NO_4$
169.14 g/mol

Benzaldehyde **7** was reduced to the corresponding alcohol **16** in a yield of 97 %.
The reaction procedure was adopted from Hu et al. [2]

*Synthesis of (5-((2,5,8,11-Tetraoxatridecan-13-yl)oxy)-2-nitrophenyl)methanol (**9**)*

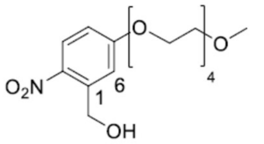

$C_{16}H_{25}NO_8$
359.38 g/mol

Oligoethylene glycol **8** (3.1 g, 8.45 mmol, 1.3 equiv.) and nitrophenol **16** (1.1 g, 6.5 mmol, 1.0 equiv.) were dissolved in dry MeCN (50 mL) and K_2CO_3 (2.7 g, 19.6 mmol, 3 equiv.) was added. The reaction was heated to reflux overnight and then filtered. The solvent was evaporated under reduced pressure. The combined organic layers were dried over $MgSO_4$ and the crude product was purified by FC (petroleum ether/ethyl acetate 1:1 to pure ethyl acetate). The product **9** was obtained as a yellow oil (1.46 g, 4.63 mmol, 71 % yield).

R_f = 0.41 (DCM/acetone 95:5).

^{1}H NMR (400 MHz, $CDCl_3$, 300 K) δ = 8.19 (d, 1H *J* = 9.2 Hz, H-3), 7.34 (d, 1H, *J* = 2.8 Hz, H-6), 6.93 (dd, 1H, *J* = 9.2 Hz, 2.8 Hz, H-4), 5.02 (d, 2H, *J* = 6.4 Hz, benzyl-CH_2), 4.29 (t, 2H, *J* = 4.7 Hz, aryl-CH_2-PEG), 3.91 (t, 2H, *J* = 4.7 Hz, aryl-CH_2-*CH_2*- PEG), 3.80-3.64 (m, 2H, CH_2), 3.73–3.62 (m, 10H, CH_2), 3.61–3.53 (m, 2H, CH_2-O-CH_3), 3.39 (s, 3H, CH_3), 2.81 (t, 1H, *J* = 6.4 Hz, OH) ppm.
^{13}C NMR (101 MHz, $CDCl_3$, 300 K) δ = 163.5 (C-5), 140.6 (C-2), 140.3 (C-1), 127.8 (C-5), 114.6 (C-6), 113.8 (C-4), 71.9 (CH_2-O-CH_3), 70.9 (CH_2), 70.6 (CH_2), 70.5 (2 x CH_2), 69.5 (CH_2-CH_2-O-aryl), 68.2 (CH_2-O-aryl), 62.8 (benzyl-CH_2), 59.0 (CH_3) ppm.
HRMS (ESI-TOF) calcd. for $C_{16}H_{25}NO_8$ [M + Na]$^+$ *m/z* = 382.1472, found: 382.1461.

*Synthesis of 13-(3-(Bromomethyl)-4-nitrophenoxy)-2,5,8,11-tetraoxatridecane (**10**)*

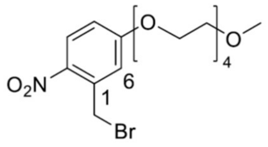

$C_{16}H_{24}BrNO_7$
422.27 g/mol

Compound **9** (200 mg, 556 µmol, 1 equiv.), CBr_4 (276 mg, 834 µmol, 1.5 equiv.) and PPh_3 (219 mg, 834 µmol, 1.5 equiv.) were dissolved in dry THF (5 mL) and stirred for 1 h. The solution was filtered. The solvent was removed under reduced pressure. The crude product was purified by FC (DCM/acetone 95:5). The product **10** was obtained as pale yellow oil (200 mg, 474 µmol, 85 % yield).

R_f = 0.49 (DCM/acetone 95:5).
^{1}H NMR (400 MHz, $CDCl_3$, 300 K) δ = 8.12 (d, 1H, *J* = 9.2 Hz, H-3), 7.06 (d, 1H, *J* = 2.8 Hz, H-6), 6.93 (dd, 1H, *J* = 9.2 Hz, 2.8 Hz, H-4), 4.48 (s, 2H, benzyl-CH_2), 4.22 (t, 2H, *J* = 4.2 Hz, aryl-CH2-PEG), 3.88 (t, 2H, *J* = 4.2 Hz, aryl-CH_2-CH_2-PEG), 3.78–3.61 (m, 10H, CH_2), 3.61–3.50 (m, 2H, CH_2-O-CH_3), 3.36 (s, 3H, CH_3) ppm.
^{13}C NMR (101 MHz, $CDCl_3$, 300 K) δ = 162.8 (*C*-5), 140.8 (*C*-2), 135.7 (*C*-1), 128.5 (*C*-3), 118.4 (*C*-6), 114.70 (C-4), 76.8 (CH2-O-CH3), 72.0 (CH_2), 71.0 (CH_2), 70.7 (CH_2), 69.4 (CH_2-CH_2-O-benzyl), 68.4 (*CH_2*-O-benzyl), 59.1 (CH_3), 29.9 (benzyl) ppm.
HRMS (ESI-TOF) calcd. for $C_{16}H_{24}BrNO_7$ [M + Na]$^+$ *m/z* = 444.0634, found: 444.0615.

*Synthesis of 1-((5-((2,5,8,11-Tetraoxatridecan-13-yl)oxy)-2-nitrobenzyl)oxy)-2,2,6,6- tetramethylpiperidine-4-carboxylic acid (**11**)*

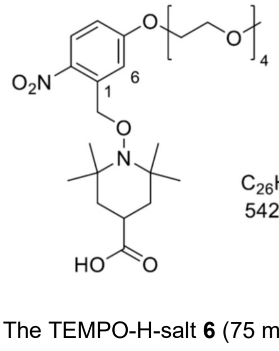

$C_{26}H_{42}N_2O_{10}$
542.63 g/mol

The TEMPO-H-salt **6** (75 mg, 282 µmol, 1.0 equiv.) was dissolved in dry, degassed THF (3 mL). 60 % NaH dispersion in mineral oil (68 mg, 1.7 mmol, 6.0 equiv.) was added. The reaction mixture was stirred for 5 min and the bromide **10** (298 mg, 705 µmol, 2.5 equiv.) was added in THF (3 mL). The reaction mixture was stirred for 1 h at room temperature. 60 % NaH dispersion in mineral oil (68 mg, 1.7 mmol, 6.0 equiv) was added and the reaction mixture was then heated to reflux for 2.5 h. The reaction was stopped by the addition of a solution of 5 % H_2O in MeOH (2 mL) and the solution was stirred

for 30 min. The solvents were then evaporated under reduced pressure. The crude product was purified by FC (ethyl acetate/acetic acid 99:1) to obtain the product **11** as a colorless oil (145 mg, 267 µmol, 95 % yield).

R_f = 0.48 (ethyl acetate:acetic acid 99:1).
^{1}H NMR (400 MHz, $CDCl_3$, 300 K) δ = 8.15 (d, 1H, J = 9.1 Hz, H-3), 7.39 (d, 1H, J = 2.8 Hz, H-6), 6.88 (dd, 1H, J = 9.1 Hz, 2.8 Hz, H-4), 5.23 (s, 2H, benzyl-CH_2), 4.24 (t, 2H, J = 4.7 Hz, aryl-CH_2-PEG), 3.91 (t, 2H, J = 4.7 Hz, aryl-CH_2-CH_2-PEG), 3.74 (m, 2H, CH_2), 3.67 (m, 8H, CH_2), 3.54 (m, 2H, CH_2), 3.37 (s, 3H, O-CH_3), 2.66 (tt, 1H, J = 13.2 Hz, 3.5 Hz, $CH_{(TEMPO)}$), 1.77 (dt, 2H, J = 13.6 Hz, 3.5 Hz, $CH_{2(TEMPO)}$), 1.68 (dt, 2H, J = 13.6 Hz, 3.5 Hz, $CH_{2(TEMPO)}$) 1.23 (s, 6H, CH_3), 1.22 (s, 6H, CH_3) ppm.
^{13}C NMR (101 MHz, $CDCl_3$, 300 K) δ = 163.2 (C-5), 139.7 (C-2), 138.6 (C-1), 127.6 (C-3), 113.4 (C-6), 112.8 (C-4), 75.6 (benzyl), 72.0 (CH_2-O-CH_3), 70.7 ($CH_{2(PEG)}$), 69.5 (CH_2-O-benzyl), 59.8 ($C_{quart.}$), 59.1 (O-CH_3), 41.9 (2x $CH_{2(TEMPO)}$), 32.8 (CH_3), 20.9 (CH_3) ppm.
HRMS (ESI-TOF) calcd. for $C_{26}H_{42}N_2O_{10}$ $[M + H]^+$ m/z = 543.2912 , found: 543.2902.

*Synthesis of Boc-4-(6-(2pyrimidinyl)-1,2,3,4-tetrazin-3-yl)benzylamine (**17**)*

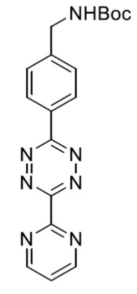

$C_{18}H_{19}N_7O_2$
365.40 g/mol

The reaction procedure was adopted from Willems et al.[3]

*Synthesis of 1-((5-((2,5,8,11-Tetraoxatridecan-13-yl)oxy)-2-nitrobenzyl)oxy)-2,2,6,6-tetramethyl-N-(4- (6-(pyrimidin-2-yl)-1,2,4,5-tetrazin-3-yl)benzyl)piperidine-4-carboxamide (**1**)*

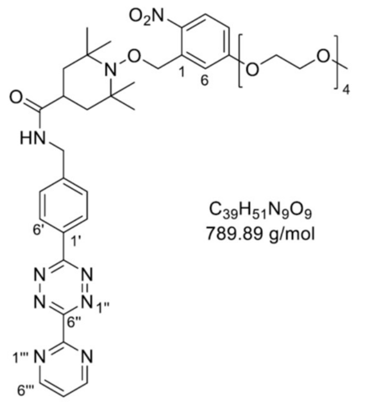

$C_{39}H_{51}N_9O_9$
789.89 g/mol

The Boc-protected tetrazine **17** (10 mg, 27 µmol, 1.0 equiv.) was dissolved in a mixture of DCM:TFA (1:1, 2 mL) and stirred for 30 min. The solvents were removed under reduced pressure to yield the free amine **12** which was used without further purification. The photo-protected spin label **11** (7 mg, 27 µmmol, 1.0 equiv.) was dissolved in dry DCM (1 mL) and PyBOP (15 mg, 30 µmol, 1.1 equiv.) was added. To this solution N-methylmorpholine (6 µL, 57 µmol, 2.1 equiv.) was added. The

solution was stirred for 5 min. Then the solution containing the spin label was transferred to the tetrazine and peptide grade, dry DMF (1 mL) was added. The solution was stirred for 3 h. The reaction mixture was diluted with DCM (4 mL) and washed with saturated $NaHCO_3$-solution (3 x 2 mL) and H2O (2 x 2 mL). The combined organic layers were dried over $MgSO_4$. The solvent was removed under reduced pressure. The crude product was purified by FC (ethyl acetate /acetone 95:5 to 93:7). The product **1** was obtained as a pink oil (16 mg, 20 µmol, 75 % yield).

R_f = 0.43 (ethyl acetate:acetone 97:3).
^{1}H NMR (400 MHz, $CDCl_3$, 300K) δ = 9.13 (d, 2H. *J* = 4.9 Hz, H-6'', H-4''), 8.69 (d, 2H, *J* = 8.1 Hz, H-6', H-2'), 8.14 (d, 1H *J* = 9.1 Hz, H-3), 7.59 (t, 1H, *J* = 4.9 Hz, H-5'''), 7.52 (d, 2H, *J* = 8.1 Hz, H-3', H-5'), 7.37 (d, 1H *J* = 2.8 Hz, H-6), 6.87 (dd, 1 H *J* = 9.1, 2.8 Hz, H-4), 6.09 (s, 1H, NH), 5.25 (s, 2H, benzyl-$CH_{2(N\text{-}O\text{-}CH2)}$), 4.58 (d, 2H *J* = 5.9 Hz, benzyl-$CH_{2(Tetrazine)}$), 4.23 (t, 2H *J* = 4.7 Hz, Aryl-O-CH_2-PEG), 3.90 (t, 2H *J* = 4.7 Hz, Aryl-O-CH_2-CH_2-PEG), 3.80 – 3.59 (m, 10H, CH_2), 3.53 (m, 2 H, CH_2), 3.35 (s, 3H, O-CH_3), 2.60 (tt, 1H *J* = 12.6, 3.5 Hz, $CH_{(TEMPO)}$), 1.92 – 1.69 (m, 4H, $CH_{2(TEMPO)}$), 1.24 (s, 12H, CH_3) ppm.
^{13}C NMR (101 MHz, $CDCl_3$, 300K) δ = 174.8 (C-carbonyl), 164.4 ($C_{quart.}$), 163.2(C-5), 163.2 ($C_{quart.}$), 159.72 ($C_{quart.}$), 158.5 (C-6''', C-4'''), 144.4 (C-5'),, 139.8 (C-2), 130.6 (C-2'''), 129.4 (C-6', C-2'), 128.7 (C-3', C-5', C-4'), 127.6 (C-3), 122.6 (C-5'''), 113.6 (C-6), 112.9 (C-4), 75.6 (C-benzyl$_{(N\text{-}O\text{-}CH2)}$), 72.1 ($CH_2$-O-$CH_3$), 71.03 ($CH_{2(PEG)}$), 70.7 ($CH_{2(PEG)}$), 70.6 ($CH_{2(PEG)}$), 69.5 (Aryl-O-$CH_2$-$CH_2$-PEG), 68.2 (Aryl-O-$CH_2$-PEG), 60.1 ($C_{quart(Tempo)}$) 59.2 (O-CH3), 43.3 (benzyl-$CH_{2(Tetrazine)}$), 42.5 (2 x $CH_{2(TEMPO)}$), 36.7 ($CH_{(TEMPO)}$), 32.6 (CH_3), 21.1 (CH_3) ppm.
HRMS (ESI-TOF) calcd. for $C_{67}H_{111}NO_{46}S$ $[M + H]^+$ *m/z* = 790.3888, found: 790.3871.

Plasmids and transformation of *E. coli*

The plasmids pEVOL_PylRS_AF and pBAD_TRX_His6_R74TAG or pBAD_TRX_His6_G34TAG_R74TAG, respectively, were used for the expression of *E. coli* thioredoxin (TRX-R74→ncAA or TRX-G34/R74→ncAA). For expression of TRX wildtype (TRX wt) only the pBAD_TRX_His6 plasmid was transformed into *E. coli*. The plasmids pEVOL_PylRS_AF and pBAD-Flag-GFP-Y39TAG-6His were used for the expression of the GFP mutant, which contains the ncAA at amino acid residue 39 instead of tyrosine (GFP-Y39→ncAA). For expression of the GFP wildtype (GFP wt) only the pBAD_Flag-GFPwt-6His plasmid was transformed into *E. coli*.
The plasmids were co-transformed into chemically competent BL21-gold (DE3) *E. coli* as follows. The reaction tube containing *E. coli* and an appropriate amount of the particular plasmid(s) was mixed by flicking and incubated 30 min on ice. The cells were then heat shocked at 42 °C for 30 sec and incubated for another 2 min on ice, before being added to 1 mL pre-warmed (37 °C) Super Optimal Broth with catabolite repression (SOC-medium). The cells were then incubated for 1 h at 37 °C and 1400 rpm, before grown on an LB-Agar plate (Lennox; ROTH) containing 34 µg/mL chloramphenicol (ROTH) and 50 µg/mL carbenicillin (ROTH) overnight.

Expression and purification of TRX and GFP and incorporation of genetically encoded artificial amino acids

For all steps performed in medium, transformed *E. coli* strains were grown in LB-medium (Lennox; ROTH), containing 34 µg/mL chloramphenicol and 50 µg/mL carbenicillin (or only carbenicillin for expression of wildtype proteins). Cells were shaken at 37 °C and 180 rpm.
TRX-R74→ncAA, TRX-G34R74→ncAA, TRX wildtype, GFP-Y39→ncAA or GFP wildtype were expressed in *E. coli* co-transformed with the respective plasmid(s) (see above) as follows. For overnight cultures, 10 mL LB-medium were inoculated with one colony of the particular *E. coli* from the agar plate. The next day, the overnight culture was diluted 1/100 (typically to a final volume of 1 L), and incubated until an OD_{600} of 0.2 – 0.3 was reached. At this point, 1 mM SCO-L-lysine (**2a**) or TCO-L-lysine (**3a**, both ncAA were bought from SICHEM) were added from freshly prepared stock solutions (therefore, the ncAA were dissolved in 0.1 M NaOH in 60 mM or 80 mM for **2a** or **3a**, respectively). Cells were further grown until an OD_{600} of 0.4 - 0.6 was reached. Protein expression was induced for 4 - 6 h with 0.2 % L-arabinose (ROTH) from a 20 % w/v stock solution. For wildtype proteins, only L-arabinose but no ncAA was used for the expression. Expression was stopped and cells were harvested by centrifugation (4 °C, 4000 rpm, 10 min). The supernatant was discarded, and the pellets were stored at -20 °C, until proteins were isolated and purified.
Proteins were purified using HisPur Ni-NTA resin (Thermo Fisher Scientific) as described elsewhere.[4] Samples were dialyzed in Slide-A-Lyzer MINI Dialysis Devices (3.5 K MWCO, Thermo Fisher Scientific) against PBS buffer, pH 7.4 (MERCK), at 4 °C. Resulting protein concentration was determined photometrically with the use of an Eppendorf BioPhotometer D30 via absorption at 280 nm (with a Factor $F_p = 1/A_{0.1\%} = 0.651$ g/L for TRX or 1.35475 g/L for GFP; $A_{0.1\%} = \varepsilon_p/MM_p$ is the absorbance of the protein at 0.1 %, ε_p is the molar extinction coefficient of the protein, and MM_p is the relative molar mass of the protein).
Integration of the ncAA and purity were confirmed by SDS-PAGE. Briefly, samples for SDS-PAGE analysis and 5 µL of a BIO-RAD Precision Plus ProteinTM Dual Color Standard were applied to a 15 % SDS-gel and run at 90 V in a BIO-RAD Mini-PROTEAN Tetra System. After Coomassie Blue staining (Brillant Blau R 250, ROTH), gels were imaged using a BIO-RAD ChemiDocTM Imaging System (Figure S11-S13).
For additional proof for expression of the correct product and for assessing fidelity of the aminoacyl-tRNA-synthetase, full-length ESI-MS spectra were recorded (Figure S16-S17).

Site-directed spin labeling of TRX and GFP with the PaNDA spin label

The labeling protocol was based on a reaction mixture of 200 µL of 40 µM protein (in PBS, pH 7.4) containing a 10-fold molar excess of PaNDA spin label (from a 10 mM stock solution in DMSO, which was stored at -20 °C). The mixture was incubated for 30 min at 20 °C and 0 rpm in an Eppendorf ThermoMixer C. The excess reactants were then removed by using HisPur Ni-NTA resin (Thermo Fisher Scientific). 120 µL of bead slurry were washed three times with MQ-H_2O in a centrifuge tube, before it was incubated together with the labeling mixture for 30 min at 4 °C under constant rotation. In a centrifuge column (Pierce Centrifuge Columns, 0.8 mL, Thermo Fisher Scientific) the beads with adherent proteins were washed approx. ten times with washing buffer (50 mM NaH_2PO_4, 300 mM NaCl, pH 8). For elution of the spin-labeled proteins, the beads were incubated for 20 min with 60 µL of washing buffer containing 500 mM imidazole, before proteins were collected by centrifugation. All wash steps and elution were performed in a benchtop centrifuge.
Wildtype proteins were also incubated with the PaNDA spin label as described above to exclude unspecific labeling (Figure S18-S19). Full-length ESI-MS of spin labeled proteins (after irradiation) proves the conversion of the ncAA-containing proteins into labeled ones (Figure S18-S19).

Deprotection of the PaNDA spin label by irradiation

A typical sample of 30 µL was filled into a glass capillary (HIRSCHMANN® ringcaps®; inner diameter 1.02 mm), and sealed with tube sealing compound (Kimble Cha-Seal) on one end. The capillary was placed on a table, and covered with a 302 nm handheld UV lamp (UVLM-28 EL Series UV Lamp, 8 Watt, 302/365 nm, analytikjena). Samples were irradiated for 2 min (or longer, if indicated; Figure S21). The sample in the capillary was directly used for subsequent EPR spectrometric measurements.

EPR measurements

EPR spectra were recorded at a BRUKER EMXnano X-band continuous wave EPR spectrometer at room temperature (approx. 22 °C). A typical sample volume of 30 µL was filled into a glass capillary (HIRSCHMANN® ringcaps®; inner diameter 1.02 mm). Spectra were recorded at a modulation amplitude of 1 G, microwave attenuation 15 dB, and a sweep width of 150 G. Typically, 20 scans of 60.06 sec scan time each were accumulated to improve the signal-to-noise ratio. Quantitative spin concentrations of samples were obtained with the use of the built-in EMXnano reference-free spin counting module (Xenon software, Bruker).
Spectra were plotted with MATLAB R2018a (The MatWorks, Inc. 3 Apple Hill Drive, Natick, MA 01760-2098, USA).

Full-length mass spectrometry of proteins

Before being subjected to mass spectrometry, the buffer of the protein samples was replaced by MQ-water in 3K spin filters (Amicon Ultra-0.5 mL Centrifugal Filters, MERCK). Protein masses were recorded by an amaZon speed ETD mass spectrometer (Bruker) with a flow rate of 4 µl/min at the Proteomics Facility of the University of Konstanz. Mass spectrometric data were evaluated using the Data Analysis Version 4.4 (Bruker) software (Figure S16-S19).

Measurement of circular dichroism (CD) spectra

Before CD measurements, the buffer of the protein samples was replaced by MQ-water using 3K spin filters. CD spectra were recorded in a JASCO J-715 Spectropolarimeter. Spectra were recorded at room temperature, using a 0.5 mm cuvette. Ten scans each were accumulated to improve signal-to-noise levels. 1000 data points were received between 280 and 180 nm. The data were baseline-corrected, and subsequently background-corrected with a sample containing only MQ-water. Noisy data at low and high wavelengths were cut off for the final presentation of the data. For the calculation of the molar residue ellipticity out of the given spectrometer unit (CD-signal in mdeg), the formula MRE = [MRW*CD-signal/1000]/(10*d*c) with M in g/mol, N_{aa} (number of amino acid residues in the protein), MRW = M/N_{aa}, c in g/mL, and d = 0.05 cm was used (Figure S20).

Experiments with *E. coli* lysate

This section describes the experiment shown in Figure 4 (main text); EPR spectral raw data of every time point are given in Figure S22. For the preparation of *E. coli* lysate 5 mL of *E. coli* overnight culture (carrying pBAD_TRX_His6 plasmids, but without expression) were prepared. After 17 hours cells were harvested by centrifugation for 10 min at 4 °C and 4000 rpm, before the supernatant was discarded. Cells were lysed with 200 µL of B-PER reagent (Thermo Fisher Scientific) containing 1 mM PMSF, and incubated for 10 min on ice with regular vortexing. The cell lysate was cleared by centrifugation for 2 min at 4 °C and 14000 rcf, and the resulting supernatant was stored on ice and further used as reducing environment for experiments. In the first part of the experiment a TRX-R74→**2b** sample was irradiated and a spin concentration of 89 µM was determined. Then 15 µL of this protein were mixed with 15 µL of the *E. coli* lysate, and EPR spectra were measured every ten min. For these experiments three scans at each time point were accumulated. The resulting plot of the spin concentration against the time showed the expected degradation kinetics of nitroxides in reducing environments. In the second part of this experiment, 15 µL of the protein, which was not irradiated in advance, were mixed with 15 µL of *E. coli* lysate, and EPR spectra were measured every twenty min. After 80 min the sample was irradiated and EPR spectra were measured every ten min to see first an increase followed by degradation of the signal (Figure 4).

Supporting Figures

NMR spectra (Figures S1-S10)

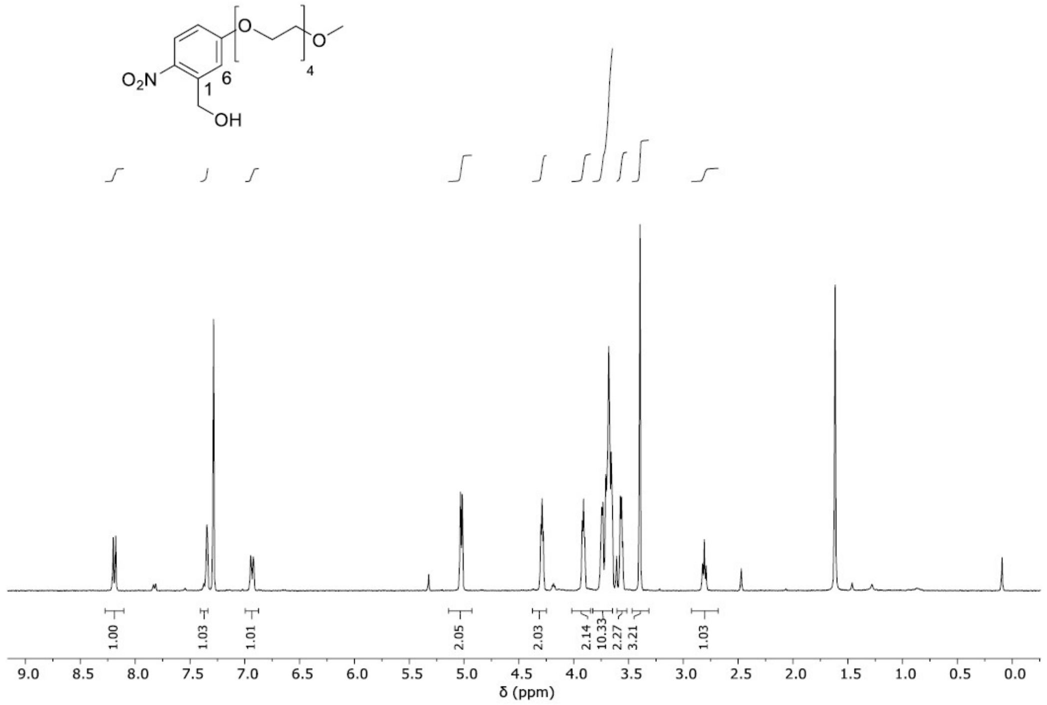

Figure S1. 1H NMR spectrum (400 MHz, CDCl3, 300 K) of compound **9**.

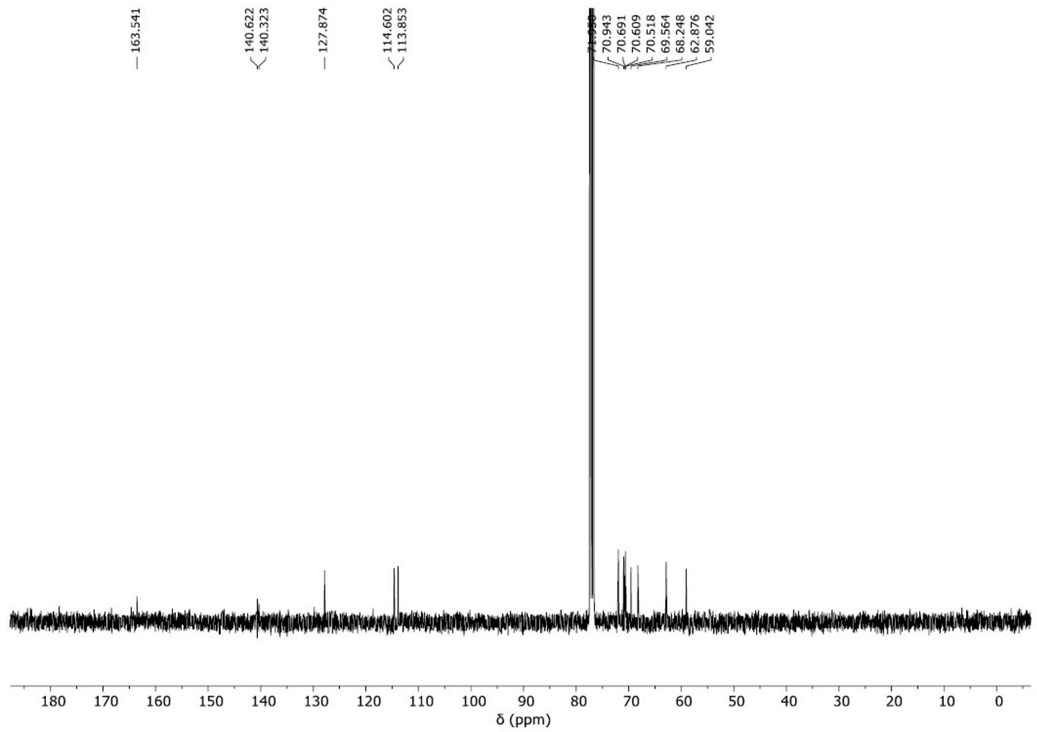

Figure S2. ^{13}C NMR spectrum (101 MHz, $CDCl_3$, 300 K) of compound **9.**

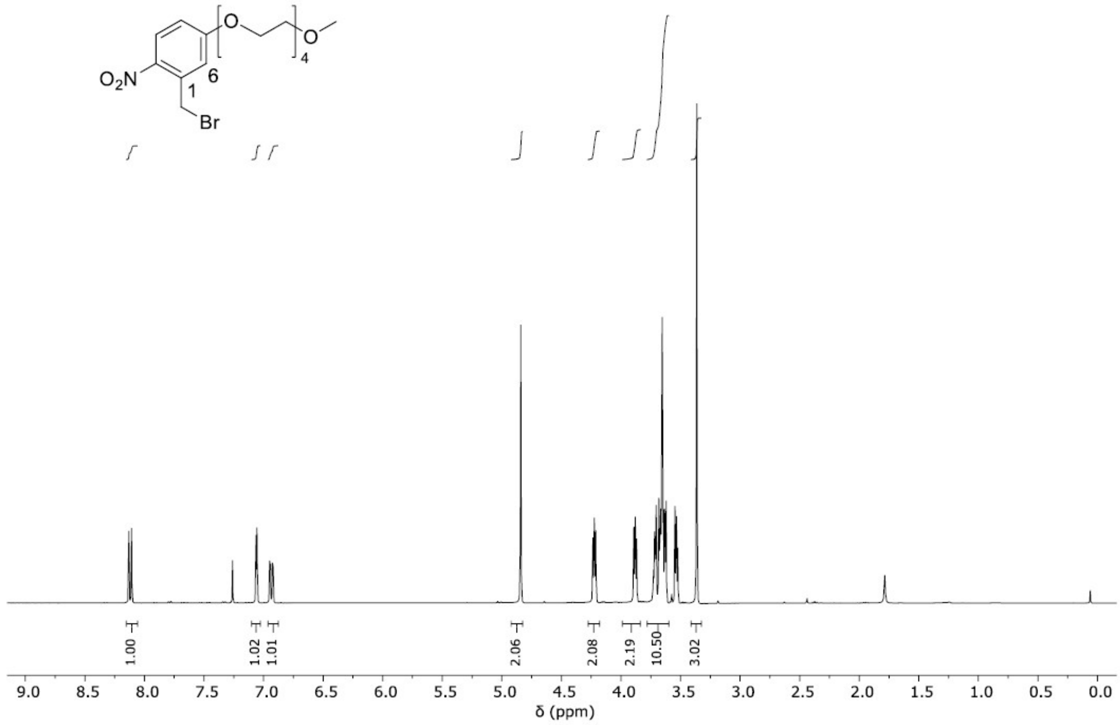

Figure S3. ^{1}H NMR spectrum (400 MHz, $CDCl_3$, 300 K) of compound **10**.

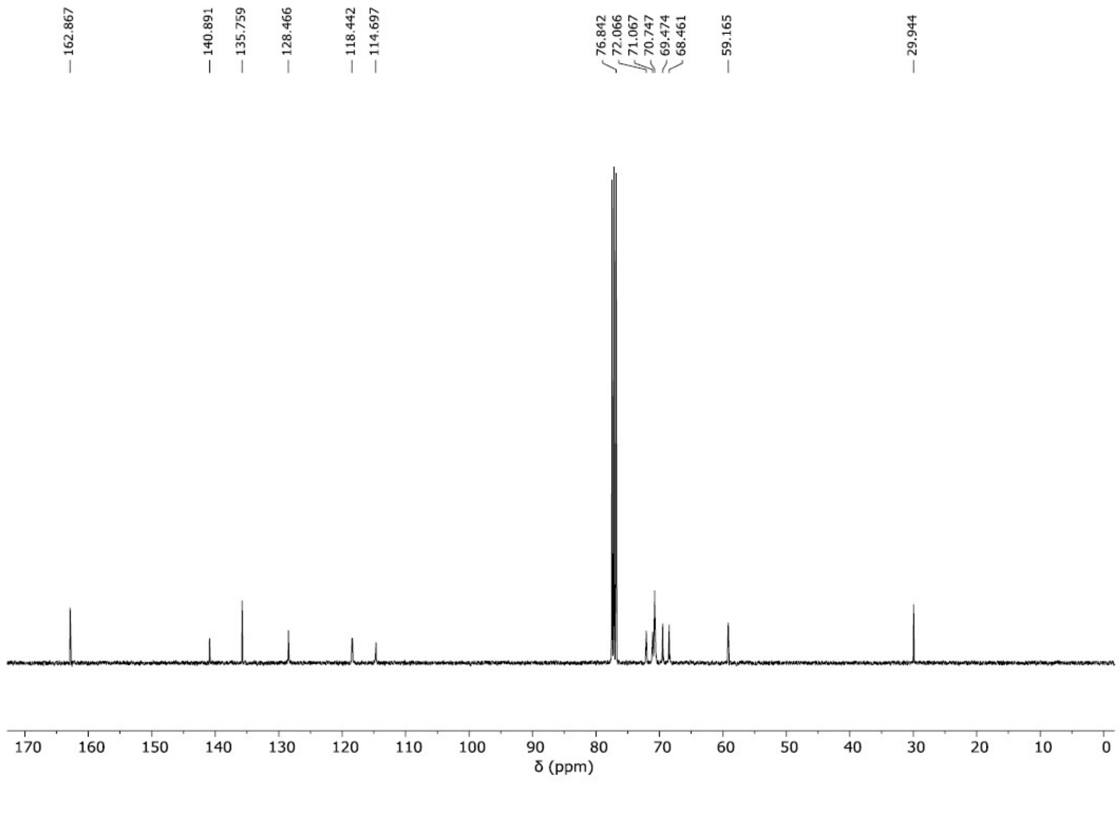

Figure S4. ^{13}C NMR spectrum (101 MHz, $CDCl_3$, 300 K) of compound **10**.

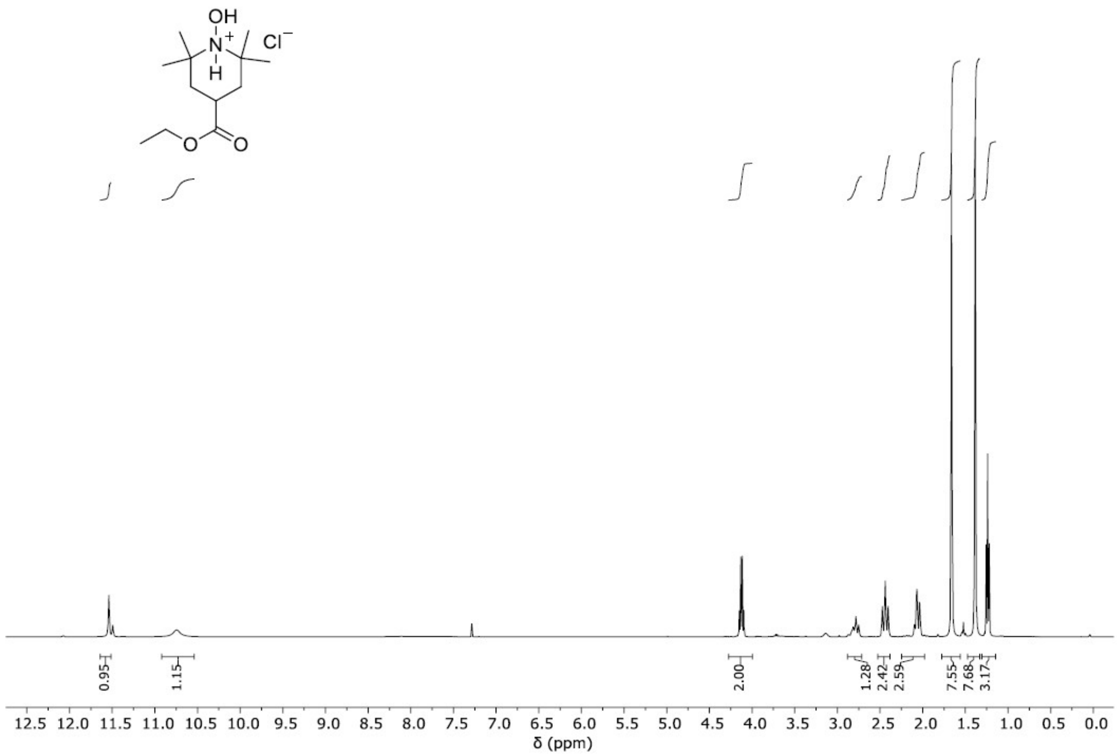

Figure S5. ^{1}H NMR spectrum (400 MHz, $CDCl_3$, 300 K) of compound **6**.

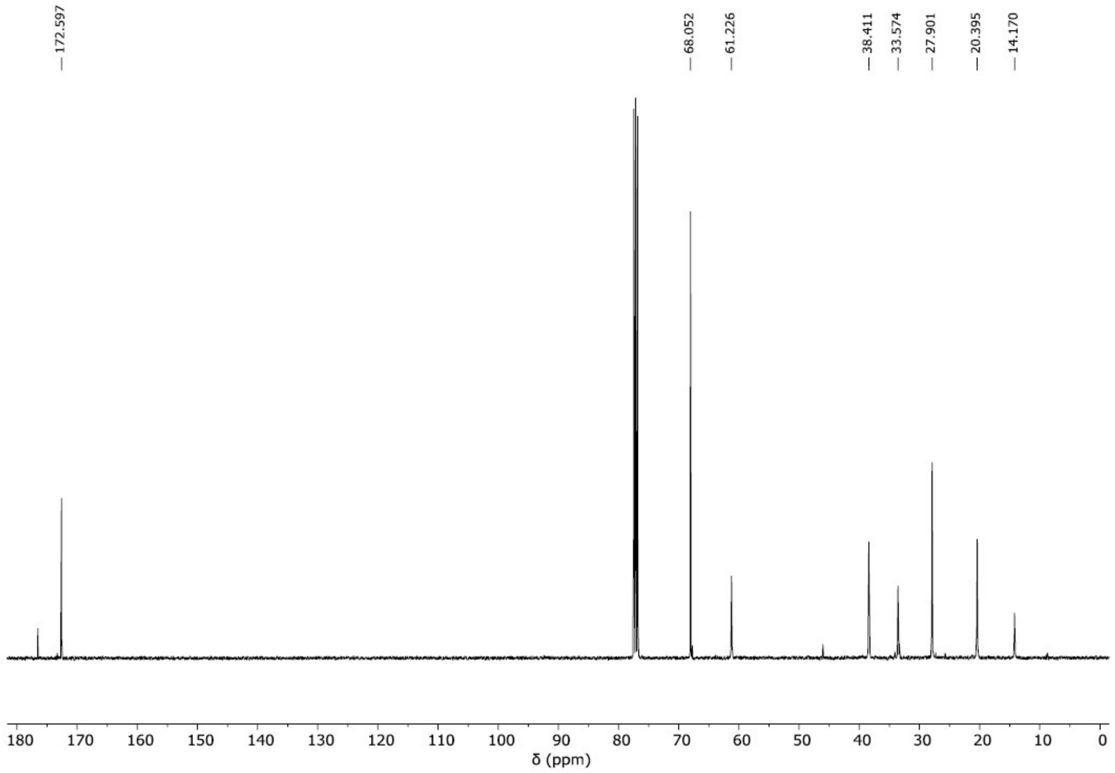

Figure S6. ^{13}C NMR spectrum (101 MHz, $CDCl_3$, 300 K) of compound **6**.

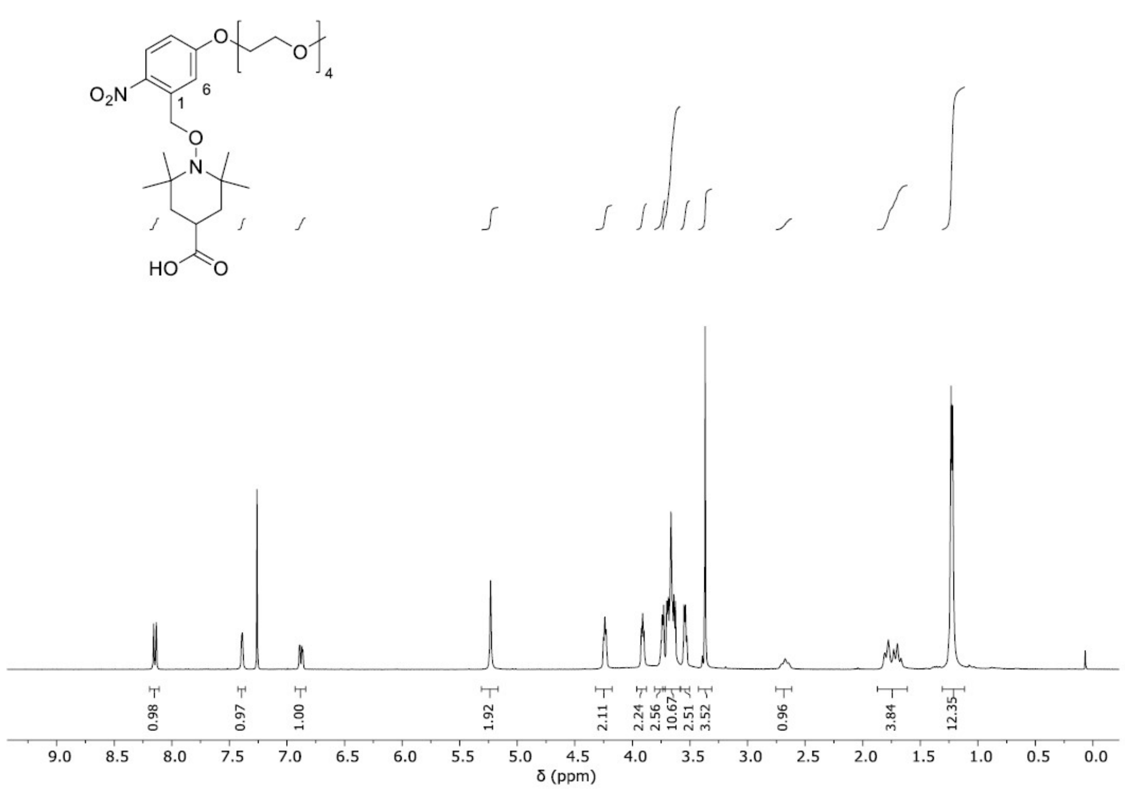

Figure S7. ^{1}H NMR spectrum (600 MHz, $CDCl_3$, 300 K) of compound **11**.

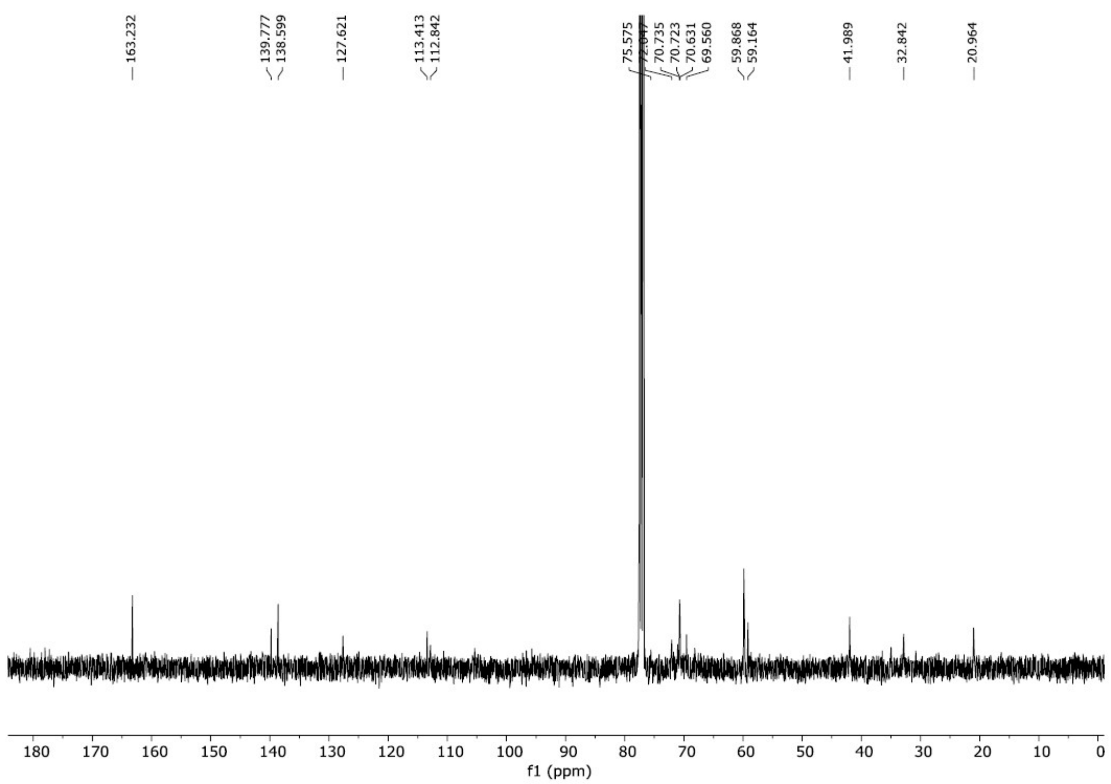

Figure S8. ^{13}C NMR spectrum (150 MHz, $CDCl_3$, 300 K) of compound **11**.

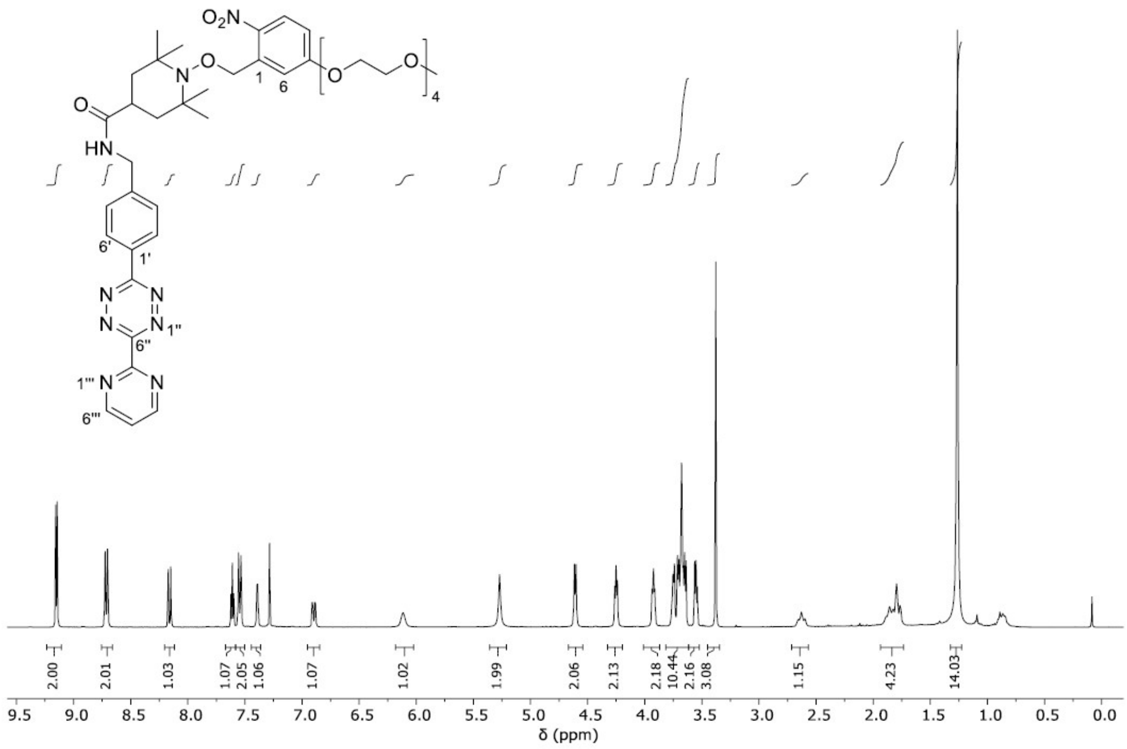

Figure S9. ^1H NMR spectrum (600 MHz, $CDCl_3$, 300 K) of compound **1**.

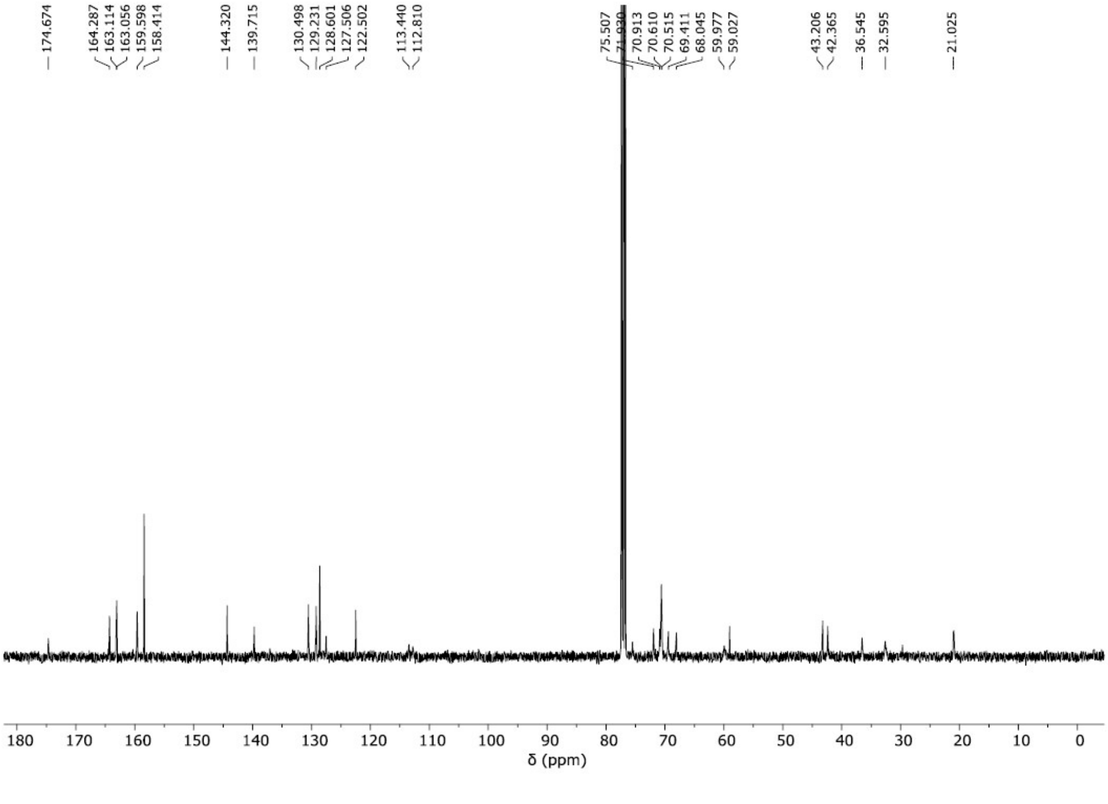

Figure S10. ^{13}C NMR spectrum (150 MHz, $CDCl_3$, 300 K) of compound **1**.

SDS-PAGE images (Figures S11-S13)

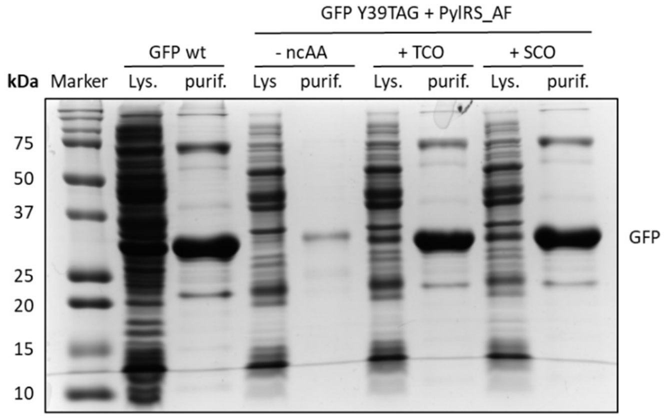

Figure S11. 15 % SDS-PAGE gel analysis of GFP wildtype and GFP-Y39→ncAA before and after purification *via* His6-tag. "-ncAA" indicates the absence of ncAA during expression.

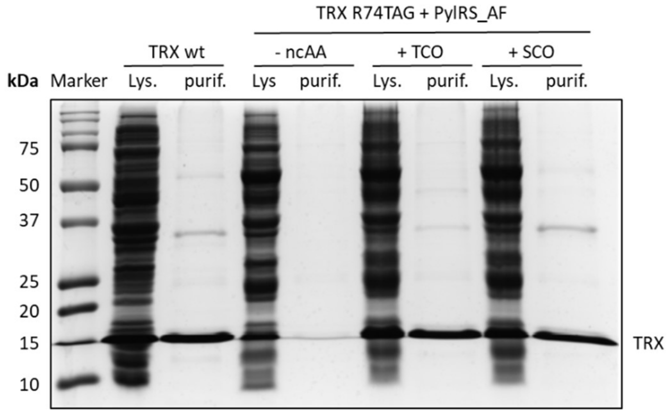

Figure S12. 15 % SDS-PAGE gel analysis of TRX wildtype and TRX-R74→ncAA before and after purification *via* His6-tag. "-ncAA" indicates the absence of ncAA during expression.

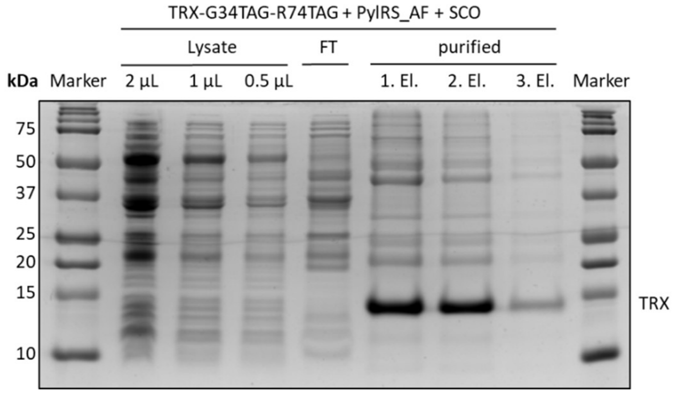

Figure S13. 15 % SDS-PAGE gel analysis of TRX-G34/R74→**2a** (**2a** = SCO-L-lysine, short: SCO) before and after purification *via* His6-tag. "FT" (flow-through) indicates the proteins not bound to Ni-NTA resin, while "1./2./3. El." indicates three subsequent elution steps of the desired protein from the Ni-NTA resin.

Primary sequences of model proteins (Figures S14-S15)

MDYKDDDDKVSKGEELFTGVVPILVELDGDVNGHKFSVSGEGEGDATYGKLTLKFICTTGKLPVPWP
TLVTTLTYGVQCFSRYPDHMKQHDFFKSAMPEGYVQERTIFFKDDGNYKTRAEVKFEGDTLVNRIELK
GIDFKEDGNILGHKLEYNYNSHNVYIMADKQKNGIKANFKIRHNIEDGSVQLADHYQQNTPIGDGPVLL
PDNHYLSTQSALSKDPNEKRDHMVLLEFVTAAGITLGMDELYKHHHHHH

Figure S14. Amino acid sequence of GFP wildtype.

MGDKIIHLTDDSFDTDVLKADGAILVDFWAEWCGPCKMIAPILDEIADEYQGKLTVAKLNIDQNPGTAP
KYGIRGIPTLLLFKNGEVAATKVGALSKGQLKEFLDANLAGSGSGERQHMDSPDLGTDDDDKHHHHH
H

Figure S15. Amino acid sequence of TRX wildtype.

Full-length ESI-MS spectra (Figures S16-S19)

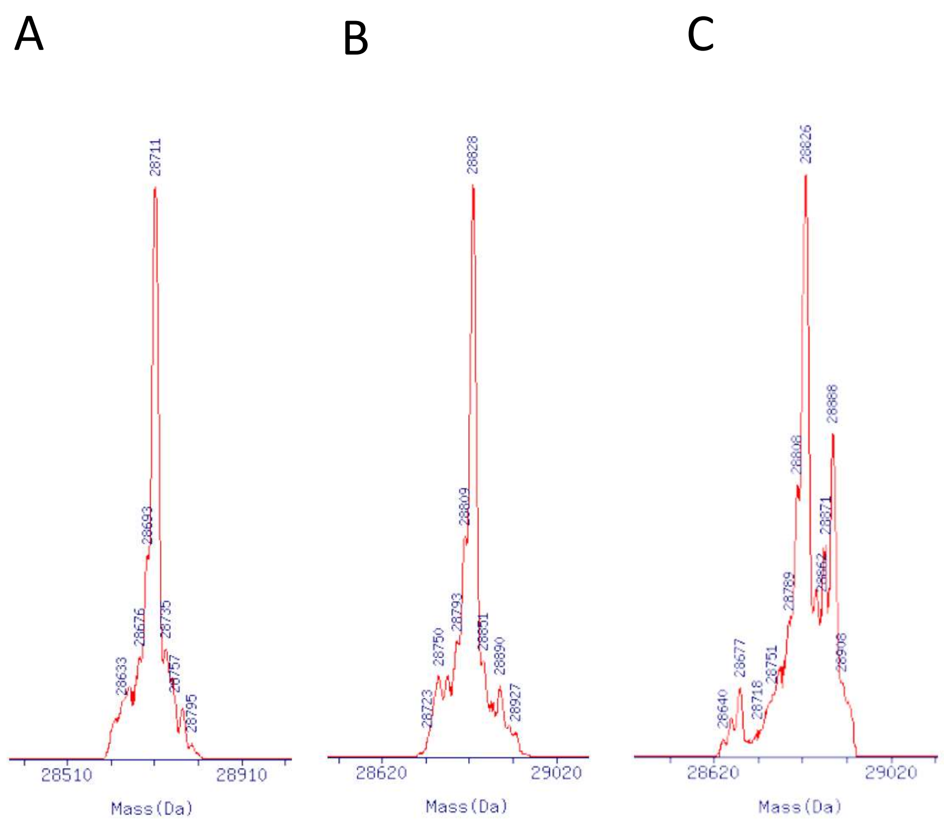

Figure S16. Full-length ESI-MS spectra of GFP samples after expression in *E. coli* and purification. A depicts the wildtype GFP, while B and C show the spectrum of GFP-Y39→**3a** or GFP-Y39→**2a**, respectively. The respective mass assigned to the desired main product is underlined.

A	28711 Da:	GFP wildtype	
B	28828 Da:	GFP-Y39→**3a**	(calcd. 28711 Da + 117 Da = 28828 Da)
C	28826 Da:	GFP-Y39→**2a**	(calcd. 28711 Da + 115 Da = 28826 Da)

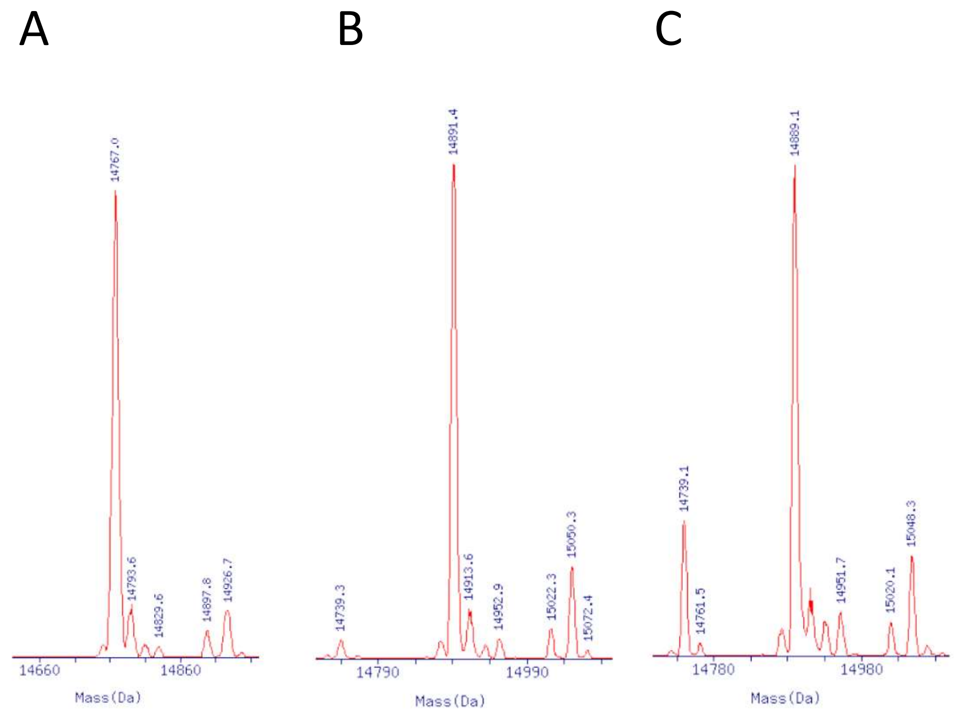

Figure S17. Full-length ESI-MS spectra of TRX samples after expression in *E. coli* and purification. A depicts the wildtype TRX, while B and C show the spectrum of TRX-R74→**3a** or TRX-R74→**2a**, respectively. The respective mass assigned to the desired main product is underlined.

A	14767 Da:	TRX wildtype	(this exact mass was found before, compare Schmidt et al.[5])
B	14739 Da:	TRX-R74→Lys	(calcd. 14891 Da – 152 Da = 14739 Da)
	14891 Da:	TRX-R74→**3a**	(calcd. 14767 Da + 124 Da = 14891 Da)
C	14739 Da:	TRX-R74→Lys	(calcd. 14889 Da – 150 Da = 14739 Da)
	14889 Da:	TRX-R74→**2a**	(calcd. 14767 Da + 122 Da = 14889 Da)

Peaks at 14739 Da indicate degradation of TCO-L-lysine **3a** or SCO-L-lysine **2a** to lysine.

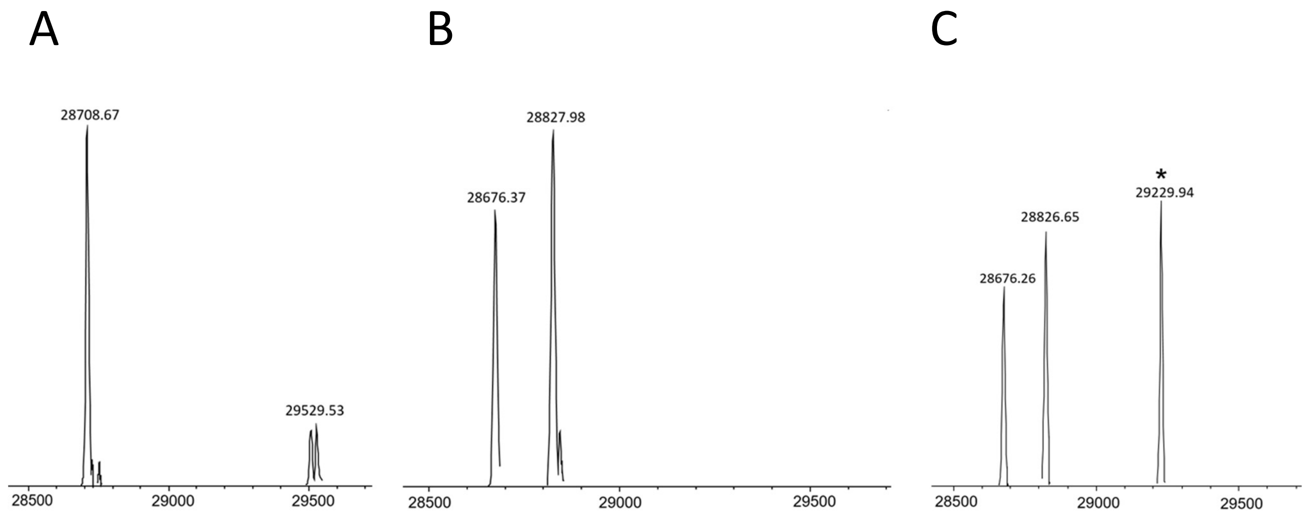

Figure S18. Full-length ESI-MS spectra of GFP samples incubated with the PaNDA spin label, then purified and irradiated. Spectrum A shows the peaks yielded after incubation of GFP wildtype with the PaNDA spin label, while B and C show the spectra after spin-labeling of the ncAA-containing GFP mutants. The respective mass assigned to the desired main product is underlined.

A	28709 Da:	GFP wildtype	(compare Figure S16)
B	28676 Da:	GFP-Y39→Lys	(calcd. 28828 Da – 152 Da = 28676 Da)
	28828 Da:	unlabeled GFP-Y39→**3a**	(compare Figure S16 B)
C	28676 Da:	GFP-Y39→Lys	(calcd. 28826 Da – 150 Da = 28676 Da)
	28827 Da:	unlabeled GFP-Y39→**2a**	(compare Figure S16 C)
	29230 Da:	labeled GFP-Y39→**2b*** after irradiation	(calcd. 28826 Da + 419 Da – 16 Da + 1 Da = 29230 Da)

The asterisk (*) indicates the elimination of the oxygen from the nitroxide, leading to the corresponding amine, due to mass spectrometry-induced fragmentation. This effect was also seen for the unbound spin label in solution (see Figure 2 B).

In the spectrum in the middle (GFP-Y39→**3a** spin labeled with the PanDA spin label) no peak can be assigned to successfully spin labeled protein (GFP-Y39→**3b**). However, in the EPR spectrum (Figure 3) successful labeling was evidenced.

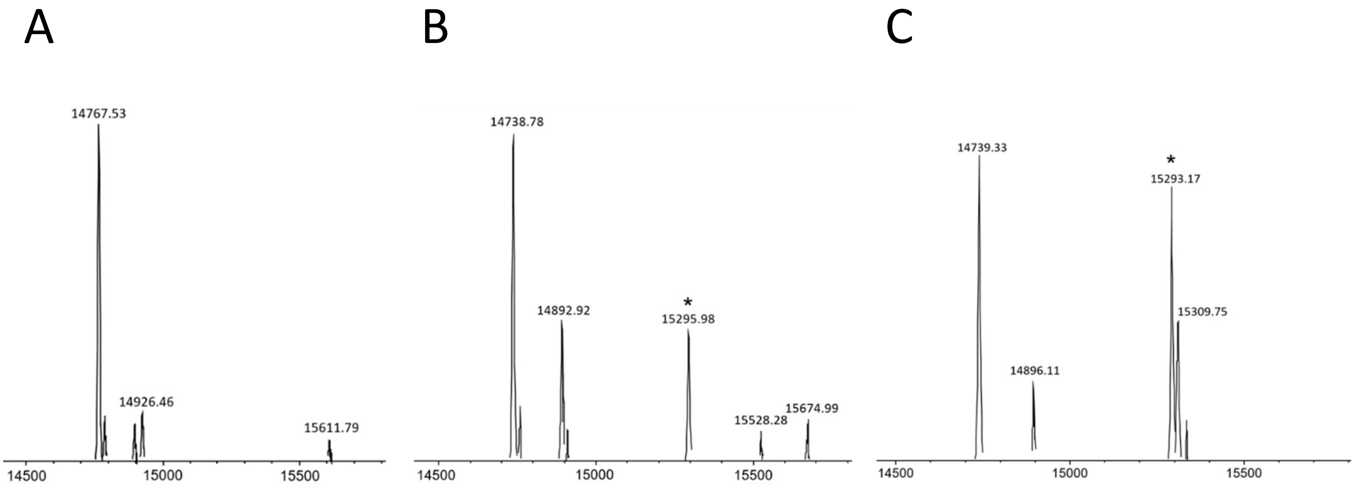

Figure S19. Full-length ESI-MS spectra of TRX samples incubated with the PaNDA spin label, then purified and irradiated. Spectrum A shows the peaks yielded after incubation of TRX wildtype with the PaNDA spin label, while B and C show the spectra after spin-labeling of the ncAA-containing TRX mutants. The respective mass assigned to the desired main product is underlined.

A	14767 Da:	TRX wildtype	(compare Figure S17 A)
B	14739 Da:	TRX-R74→Lys	(compare Figure S17 B)
	14893 Da:	unlabeled TRX-R74→**3a**	(compare Figure S17 B)
	15296 Da:	labeled TRX-R74→**3b*** after irradiation	(calcd. 14893 Da + 419 Da – 16 Da + 1 Da = 15297 Da)
C	14739 Da:	TRX-R74→Lys	(compare Figure S17 C)
	15293 Da:	labeled TRX-R74→**2b*** after irradiation	(calcd. 14889 Da + 419 Da -16 Da + 1 Da = 15293 Da)
	15310 Da:	labeled TRX-R74→**2b** after irradiation	(calcd. 15294 Da - 1 Da + 16 Da = 15309 Da)

The asterisk (*) indicates the elimination of the oxygen from the nitroxide, leading to the corresponding amine, due to mass spectrometry-induced fragmentation. This effect was also seen for the unbound spin label in solution (see Figure 2 B).

Further supplementary figures (Figures S20-S22)

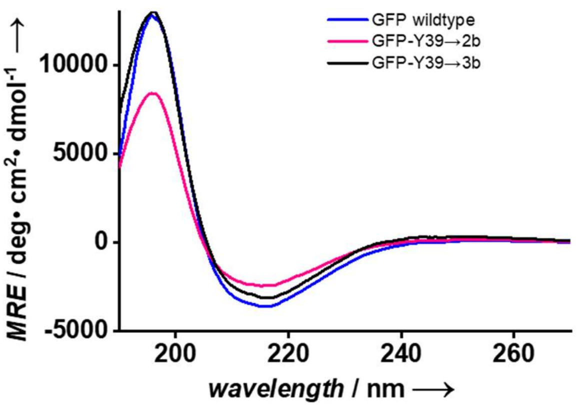

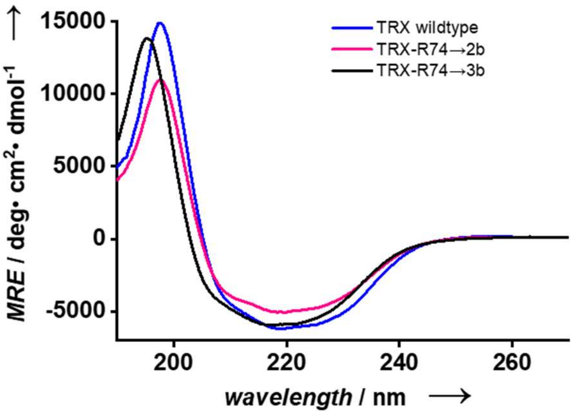

Figure S20. CD analysis of GFP (left) and TRX (right). Wildtype proteins (blue line) were measured after expression (without labeling), while the red and black lines refer to labeled proteins. All samples were desalted before measurement, and spectra were baseline- and background-corrected. The qualitative spectral shape is found to be unchanged. Quantitative deviations upon labeling are due to limited accuracy of the protein concentration determination.

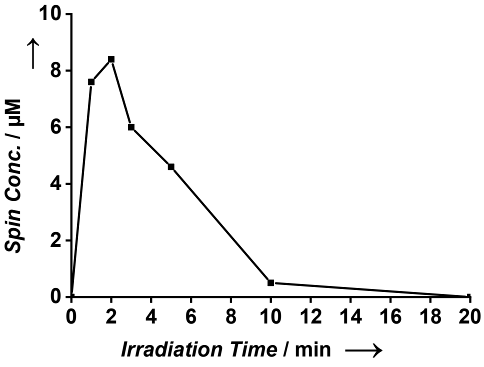

Figure S21. Evaluation of the deprotection kinetics of the photocage group of the PaNDA spin label attached to a protein. Each time point stems from an individual TRX-R74→**2a** sample, that was labeled with the PaNDA spin label as described above (TRX-R74→**2b**), and irradiated at 302 nm for the indicated durations. The maximum indicates, that irradiation for 2 min yielded the highest deprotection.

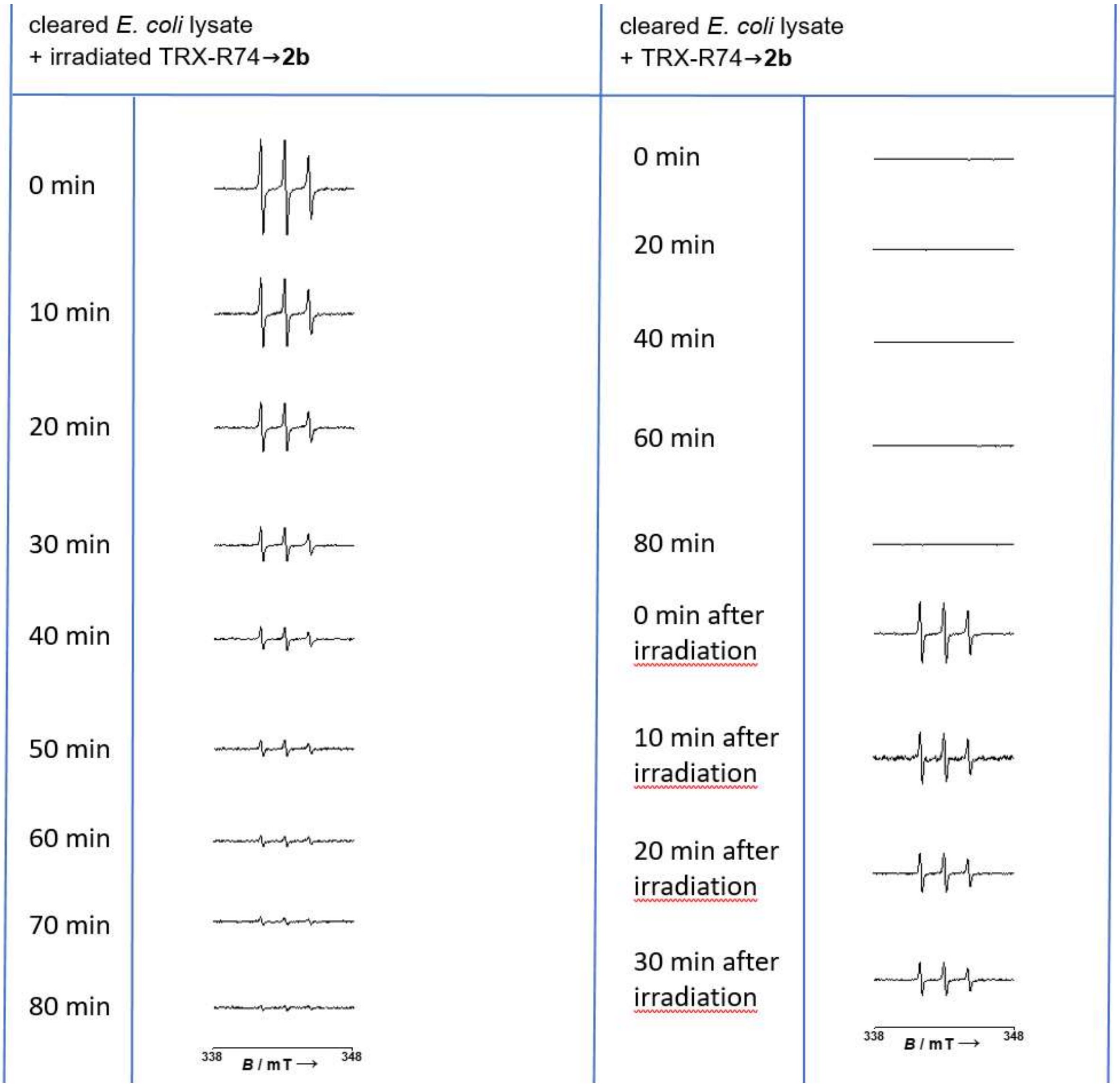

Figure S22. EPR spectra of the experiment shown in Figure 4 (main text). All spectra are drawn on the same y-axis range. The spectrum "10 min after irradiation" shown in the 2nd column on the right was measured with 25 dB microwave attenuation instead of 15 dB, leading to a higher signal-to-noise ratio (SNR).

Supporting References

[1] Z. Wang, K. Zhong, Y. Liang, T. Chen, B. Yin, M. Lee, L. Y. Jin, *J. Polym. Sci., Part A: Polym. Chem.* **2015**, *53*, 85.
[2] X. Hu, J. Shi, S. W. Thomas, *Polym. Chem.* **2015**, *6*, 4966.
[3] L. I. Willems, N. Li, B. I. Florea, M. Ruben, G. A. van der Marel, H. S. Overkleeft, *Angew. Chem., Int. Ed.* **2012**, *51*, 4431.
[4] M. J. Schmidt, A. Fedoseev, D. Summerer, M. Drescher, *Methods Enzymol.* **2015**, *563*, 483.
[5] M. J. Schmidt, J. Borbas, M. Drescher, D. Summerer, *J. Am. Chem. Soc.* **2014**, *136*, 1238.
